# Thermodynamic and hydrochemical controls on CH_4_ in a coal seam gas and overlying alluvial aquifer: new insights into CH_4_ origins

**DOI:** 10.1038/srep32407

**Published:** 2016-08-31

**Authors:** D. Des. R. Owen, O. Shouakar-Stash, U. Morgenstern, R. Aravena

**Affiliations:** 1 School of Earth, Environmental and Biological Sciences, Queensland University of Technology, Brisbane, Queensland, 4000, Australia; 2 Isotope Tracer Technologies, Waterloo, ON N2V 1Z5, Canada; 3 GNS Science, Lower Hutt 5014, P.O. Box 30368, New Zealand; 4 Department of Earth and Environmental Sciences, University of Waterloo, Ontario N2L 3G1, Canada

## Abstract

Using a comprehensive data set (dissolved CH_4_, δ^13^C-CH_4_, δ^2^H-CH_4_, δ^13^C-DIC, δ^37^Cl, δ^2^H-H_2_O, δ^18^O-H_2_O, Na, K, Ca, Mg, HCO_3_, Cl, Br, SO_4_, NO_3_ and DO), in combination with a novel application of isometric log ratios, this study describes hydrochemical and thermodynamic controls on dissolved CH_4_ from a coal seam gas reservoir and an alluvial aquifer in the Condamine catchment, eastern Surat/north-western Clarence-Moreton basins, Australia. δ^13^C-CH_4_ data in the gas reservoir (−58‰ to −49‰) and shallow coal measures underlying the alluvium (−80‰ to −65‰) are distinct. CO_2_ reduction is the dominant methanogenic pathway in all aquifers, and it is controlled by SO_4_ concentrations and competition for reactants such as H_2_. At isolated, brackish sites in the shallow coal measures and alluvium, highly depleted δ^2^H-CH_4_ (<310‰) indicate acetoclastic methanogenesis where SO_4_ concentrations inhibit CO_2_ reduction. Evidence of CH_4_ migration from the deep gas reservoir (200–500 m) to the shallow coal measures (<200 m) or the alluvium was not observed. The study demonstrates the importance of understanding CH_4_ at different depth profiles within and between aquifers. Further research, including culturing studies of microbial consortia, will improve our understanding of the occurrence of CH_4_ within and between aquifers in these basins.

Methane (CH_4_) is a ubiquitous substance that occurs in adsorbed, dissolved and free gas forms in a range of aquifer, surface water, soil and atmospheric environments^1^,^2^. In surface waters and the shallow subsurface, CH_4_ production and consumption are mediated by microbial processes that are stimulated by changes in redox conditions, availability of suitable fermentation substrates and electron acceptors^2^,^3^,^4^. The relative abundance of heavy and light stable isotopes of carbon (^12^C/^13^C) and hydrogen (^2^H/^1^H) that comprise CH_4_ is influenced by certain processes including: the type of methanogenic (production) and consumption pathways; transport processes such as diffusion and desorption; competition for substrates with other reducing organisms (e.g. SO_4_-reducers); and thermodynamic conditions^3^,^5^,^6^,^7^,^8^,^9^,^10^,^11^,^12^. As a result, isotopic data of CH_4_ can be ambiguous, particularly when interpreting data from larger scales, where multiple sources of CH_4_ exist, where CH_4_ has potentially moved or where thermodynamic conditions change. There has been a range of research dedicated to understanding these complexities^3^,^4^,^5^,^6^,^8^,^13^,^14^,^15^,^16^. Yet, while information on the complex behaviour of CH_4_ isotopes is readily available, it creates some uncertainty about the value of CH_4_ isotopes as indicators of broader processes, such as fugitive emissions or aquifer connectivity (see Figure S1 for examples of δ^13^C-CH_4_ values under a range of different pathways and conditions).

Recent rapid development of unconventional gas resources such as coal seam gas (coal bed methane) or shale gas (CH_4_ sorbed under pressure in coal measures or shale deposits) has spurred interest in understanding the extent of unconventional gas resources and the potential for gas migration within and between aquifers. Dissolved gas can migrate within and between aquifers either via advection or by a diffusion process^12^,^17^. Some previous research in shale-gas-bearing basins has shown that CH_4_ can migrate with brines from underlying gas-bearing aquifers but, in the absence of hydrocarbon reservoirs, it can also be generated *in situ*^18^,^19^,^20^.

This paper investigates origins and transport of dissolved CH_4_ in a hydrogeological setting where a shallow coal seam gas (CSG) reservoir underlies an important alluvial water resource (Condamine River catchment, Surat and Clarence-Moreton basins, Australia). We use a novel approach, employing a combination of hydrochemical, CH_4_ and isotope data with isometric log ratios (reactants and products) and Gibbs free energy calculations from key biological reaction processes to describe the thermodynamic constraints on CH_4_ in the alluvium and underlying coal measures. This approach addresses the complexity of CH_4_ production and consumption in the subsurface and the range of associated isotopic responses. The approaches/techniques used to address this complexity are outlined in Table 1. Previous interpretations of δ^13^C-CH_4_ data from a similar area^21^ are compared to new data presented here and conclusions regarding CH_4_ migration are reviewed. A minimum suite of parameters required to assess CH_4_ within and between aquifers is proposed for future monitoring and data collection.

## Hydrogeological Setting

**Figure 1:**
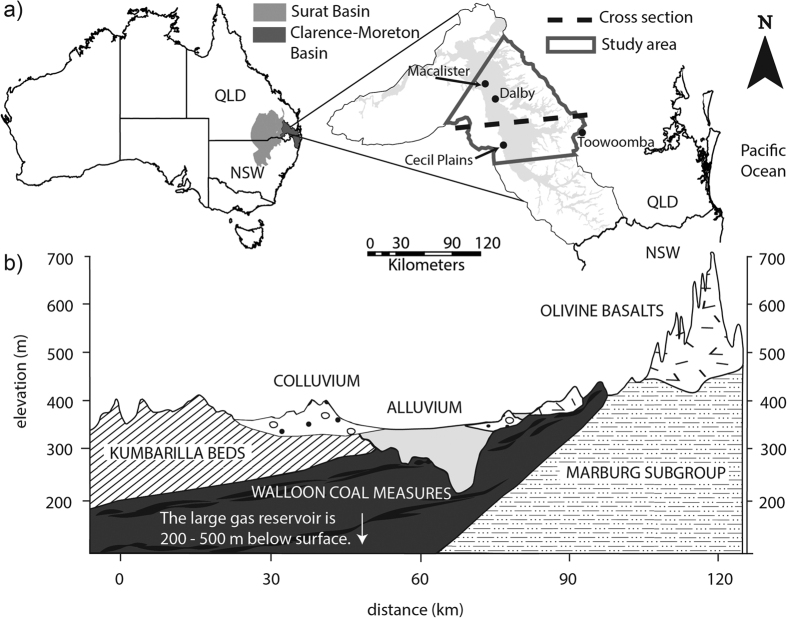
Hydrogeological setting and study area, showing: (**a**) location of the Condamine River catchment and Surat/Clarence-Moreton basins in eastern Australia; and (**b**) conceptual cross section of the Condamine alluvium and adjacent sedimentary features. For (**a**), the basin boundary is defined as the Kumbarilla Ridge^28^ ^and references therein^^30^. (**a**) was prepared using *ArcGIS v 10.1* (www.esri.com) and modified in *Adobe Illustrator CC 2014*. For (**b**), the land surface and alluvial depth profile is real, as taken from the Condamine Groundwater Visualisation System (GVS)^38^: the outcrops and depth extent of the olivine basalt and sedimentary bedrock features have not been mapped in detail and are represented as conceptualisations based on interpretations of existing literature^22^,^25^,^39^ by the co-authors.

The Condamine River alluvium (the Condamine alluvium) occurs in the Condamine River catchment, which is a large subcatchment (30,451 km^2^) in the headwaters of the Murray-Darling Basin in southeast Queensland, Australia. This study focusses on the upper, central Condamine alluvium (Fig. 1a). Hydrogeology and hydrochemistry of the alluvium are summarised in previous published work^22^,^23^,^24^. The alluvium overlies the Walloon Coal Measures (the coal measures) and, on the western alluvial flank, parts of the Kumbarilla Beds, which are Jurassic sedimentary features of the Surat and Clarence-Moreton basins (Fig. 1b). At the bedrock-alluvial interface an impervious clay layer (termed: the “transition layer”) is proposed to limit interaction with the underlying coal measures; however, the spatial extent of this transition layer is not well known. In some cases, the alluvium has incised the coal measures by up to 130 m within a paleovalley (QWC^25^) (Fig. 1c). Weathered bedrock materials, including coal fragments, occur throughout the alluvium^22^. The alluvium is exploited for water reserves for use in large-scale irrigation, mainly cotton. Higher quality water is generally found in upstream areas of the study area, near Cecil Plains where hydraulic conductivity is higher^22^.

Coal seam gas (CSG) reserves in the underlying coal measures are a significant economic resource and gas production in the study area is focussed on areas in the south west at depths of ~300–500 m, (see Fig. 1). Production of coal seam gas requires water to be extracted from the coal seam which has raised concerns about aquifer connectivity. The CH_4_ gas in the gas reservoirs is typically biogenic, it tends to be concentrated at geological structures, and coal seams are discontinuous^26^,^27^,^28^,^29^,^30^,^31^. Using δ^13^C-CH_4_ of free gas that was collected from degassing alluvial wells during pumping, a recent study concluded that CH_4_ leakage from the coal measures to the alluvium was occurring in some areas and this was used to infer aquifer connectivity^21^. However, this study did not collect any CH_4_ data (free or dissolved) from the underlying coal measures for reference. A previous study that examined δ^7^Li within and between coal measure and basalt aquifers found very low concentrations of Li in the alluvium when compared to the coal measures^32^. Assuming conservative behaviour of the Li ion^33^,^34^,^35^,^36^,^37^, these results suggest large-scale solute transport between these aquifers is not occurring. While the gas reservoir that underlies the Condamine catchment is relatively shallow compared to some other areas in the Surat Basin, the commercially viable gas reservoir that directly underlies the Condamine catchment is relatively deep (typically 300–500 m) when compared to the maximum alluvium depth (130 m). In this paper we refer to two areas of the coal measures as follows: (1) the *CSG or gas reservoir* (200–500 m) of the coal measures where commercial gas reserves are found; and (2) the *shallow coal measures*: shallower zones of the coal measures (<200 m) that are up gradient of the gas reservoir, but which are underlying or adjacent to the alluvium (see Fig. 1b).

## Results and Discussion

### Redox and salinity conditions

The deep gas reservoir is characterised by highly reduced SO_4_ (typically less than detection limit (DL) (1 mg/L, or 0.02 meq/L)), and brackish water (Cl = 1000–4500 mg/L or 28–127 meq/L). In the shallower coal measures the SO_4_ and Cl concentrations are more variable and show a positive relationship, ranging from <0.1 mg/L to 488 mg/L (10 meq/L) for SO_4_ and 82 mg/L (2.3 meq/L) to 4680 mg/L (131.8 meq/L) for Cl. The majority of shallow coal measure samples have SO_4_ concentrations below 50 mg/L (1 meq/L). A single coal sample from the shallow coal measures underlying the alluvium at Cecil Plains showed small amounts of pyrite; however, SO_4_ concentrations at this site ranged from 8–12 mg/L (0.16–0.25 meq/L), indicating that SO_4_ is not completely reduced at this site. Salinity in the alluvium is also highly variable (Cl ranging from 35–8700 mg/L, or 1–245 meq/L) and also shows a positive relationship with SO_4_, which ranges from <1 mg/L to 988 mg/L (20 meq/L). Peak Cl and SO_4_ concentrations are found in shallow (~20 m) wells. This is consistent with the findings of Owen and Cox^23^, which showed higher salinity is related to evapotranspiration processes.

NO_3_ is low in all aquifers: typically <0.05 mg/L (0.001 meq/L), with 8 samples having NO_3_ below DL (0.01 mg/L or 0.00016 meq/L) for the shallow coal measures, and ranging from 0.02 mg/L (3.23e-04 meq/L) to 2.3 mg/L (3.64e-02 meq/L), with three samples below DL (0.01 mg/L or 1.61e-04 meq/L), for the alluvium. With the exception of one shallow coal measure sample underlying a basalt outcrop (P19) and a shallow (~27 m) alluvial well (ID GM1076), all samples that contained CH_4_ had NO_3_ concentrations below 0.006 meq/L (0.37 mg/L) which is favourable for methanogenesis^38^. We found no NO_2_ above DL (0.01 mg/L) in any aquifer: this shows that significant denitrification is not occurring in these aquifers.

Data on dissolved Fe^2+^/Fe^3+^ and Mn species were not available, however, total dissolved concentrations of these ions were low in all aquifers in the study area. In the alluvium, Fe above DL (0.05 mg/L) was found at only 5 sites, (0.11–4.86 mg/L), while Mn concentrations were above DL (0.001 mg/L) at only 12 sites, the majority of which had Mn concentrations <0.01 mg/L. In the shallow coal measures, 8 samples had Fe above DL, with 6 of these being <0.8 mg/L, while only Mn concentrations were <0.09 mg/L at the majority (n = 12) of sites. These low values compare with production water which is highly reduced (SO_4_ < 1 mg/L) (see Supplementary Information Table S1).

### Tritium

Tritium analyses (DL = 0.02 TU) were performed at selected sites: shallow coal measures (n = 5) and alluvium (n = 9). Significant ^3^H was observed for only one shallow coal measures well (P12, 0.95 TU): this well occurs on the basalt ranges under a thin basalt outcrop in a recharge area and contained no CH_4_. Only 2 shallow alluvial wells (~27 m (GM1076) and 41 m (GM1338)) were found to have detectable^3^ (0.05 TU and 0.22 TU, respectively): these were located ~6 and 16 km from the river, respectively. Only one of these wells contained CH_4_ (GM1076: 0.05 TU). Alluvial wells with no detectable tritium ranged from 18 m–89 m in depth, including 4 wells <40 m. The absence of tritium in the majority of shallow wells indicates limited to no modern recharge. While river recharge is considered important in this alluvial system^24^, we found no tritium in a 57 m deep alluvial well (GM0057) located approximately 1.4 km from the river.

### Stable isotopes of chlorine (δ^37^Cl)

δ^37^Cl was measured for all samples containing CH_4_ within and between aquifers to provide an additional parameter for understanding possible CH_4_ migration via a diffusion pathway. δ^37^Cl for these samples ranged from −2.52‰ to −0.1‰ in the CSG reservoir; −1.11‰ to 0.8‰ in the shallow coal measures; and −0.72‰ to 0.89‰ in the alluvium.

### Dissolved organic carbon

Dissolved organic carbon (DOC) is typically low in coal measures and alluvium aquifers, ranging from 0.3–1.6 mg/L, and 0.1–3.9 mg/L, respectively. The shallowest well (GM1073: 18 m) had the second highest DOC in the alluvial data set 0.4 mg/L, although DOC of all alluvial and coal measure samples could be considered low compared to other studies^19^,^39^,^40^. We found no relationship between DOC and CH_4_ concentrations in either aquifer.

The two alluvial wells that contained detectable tritium (GM1076 and GM1338) contained 0.2 and 0.4 mg/L of DOC, respectively. Two wells that occur at the alluvial-coal measure interface had DOC concentrations of 0.4 mg/L (GM1193:110 m) and 7 mg/L (IND2:85 m). The shallower sample contained no CH_4_ and presented as an anomaly in the dataset. δ^7^Li for this sample^32^ and lithological analysis confirm it is in the upper layer of the coal measures (~7 m below the alluvial basement). The other sample at the alluvial-coal measure transition zone (110 m) contained CH_4_ and occurs in an area where the alluvium appears to have incised the coal measures; drill logs show it contains both sand and coal fragments.

### CH_4_ within and between aquifers

Dissolved CH_4_ concentrations in the deep CSG reservoir ranged from 2000 μg/L–25000 μg/L (n = 21). In total, 7 of the 14 shallow coal measure wells contain dissolved CH_4_ above DL (10 μg/L): concentrations ranged from 95–18000 μg/L. Of the 23 wells sampled in the alluvium, only 5 were found to contain CH_4_, with concentrations ranging from 10–535 μg/L. All alluvial samples with dissolved CH_4_ were found in monitoring wells which were sampled using low flow techniques.

In this study, CH_4_ is predominantly the sole hydrocarbon above DL (10 μg/L), with only 2 samples in the shallow coal measures (IND1 and IND3) containing small concentrations of ethane and propane above DL (10 μg/L).

**Figure 2:**
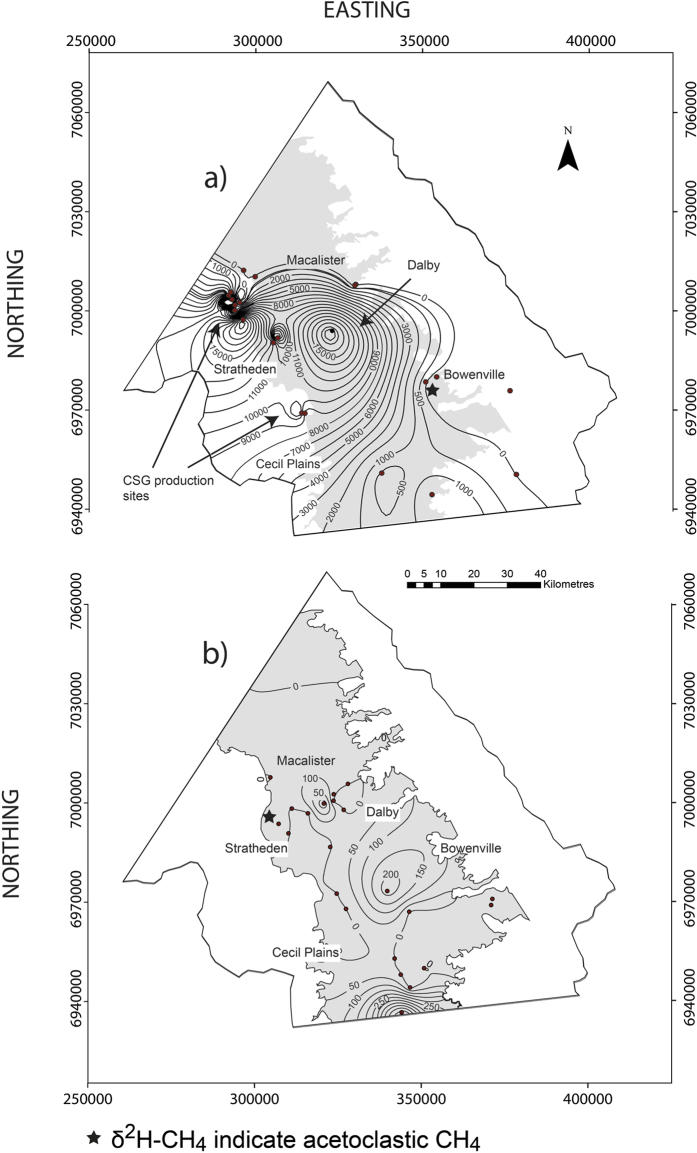
CH_4_ μg/L contours for: (**a**) the coal measures, including the gas reservoir and the shallow coal measures; and (**b**) the alluvium. CSG production sites are marked by arrows in (**a**). Points in (**a**,**b**) represent sample locations. Maps were prepared using *ArcGIS v 10.1* and modified using *Adobe Illustrator CC 2014.* Contours were determined using the spline tool in *ArcGIS v 10.1* (www.esri.com), which interpolates a raster surface from points using a two-dimensional, minimum curvature spline technique and which passes a contour line through each measured point. Contours marked as 0 are based on measured CH_4_ below DL (<10 μg/L) at relevant points. This representation of CH_4_ μg/L distribution should be used for conceptual/visualisation purposes for this data set only, as methanogenic and methanotrophic conditions may only be favourable at discrete locations and because contours between points represent a conceptual change in the concentration gradient between measured samples only.

Figure 2a,b conceptualise the spatial distribution of CH_4_ in the shallow coal measures and the alluvium, respectively. The dissolved CH_4_ in the alluvium occurred over a large depth range (~20–110 m) and over a large spatial area: CH_4_ distribution in the alluvium is relatively sparse, and peak alluvial-CH_4_ concentrations do not show any spatial relationship with peak CH_4_ concentrations in the underlying coal measures.

### δ^13^C-CH_4_ and δ^2^H-CH_4_ within and between aquifers

While thermogenic methane typically has more enriched δ^13^C values, biogenic CH_4_ can also have δ^13^C values within what is considered a typical thermogenic range. For example, the dominance of acetoclastic methanogenesis^2^,^5^,^13^,^26^,^41^,^42^, shifts in seasonal availability of the substrate^43^,^44^, enrichment of the CO_2_ pool as a result of on-going methanogenesis^3^, and anaerobic (AOM) or aerobic oxidation of CH_4_^8^,^45^,^46^ can all produce CH_4_ that is relatively enriched in δ^13^C (see Figure S1 for a summary). Fractionation factors can offer insights into production and consumption pathways: α_DIC-CH4_ of ~1.07 and α_H2O-CH4_ of ~1.2 are typically indicative of CO_2_ reduction pathways, while of α_DIC-CH4_ ~1.04 and α_H2O-CH4_ ~1.4 are typically indicative of acetoclastic methanogenesis or an oxidation pathway^3^,^5^,^47^.

**Figure 3:**
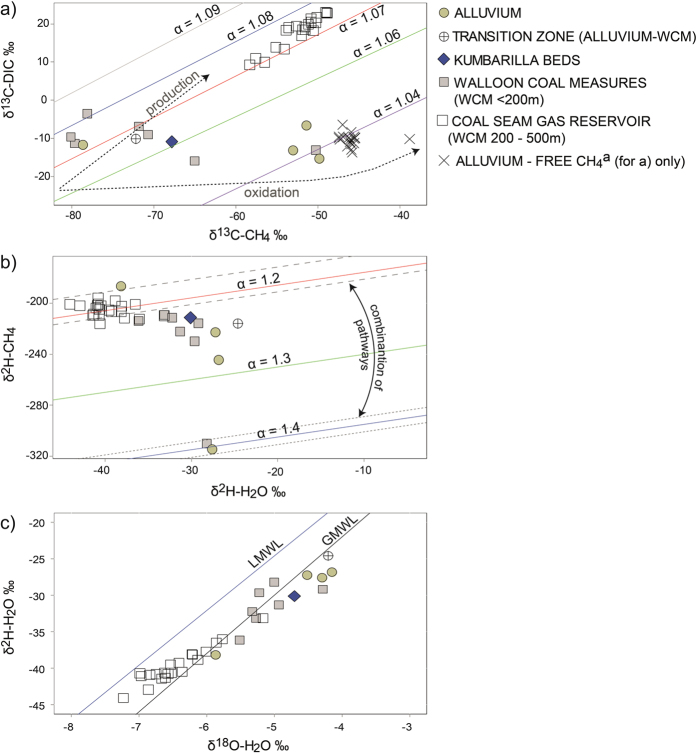
Comparisons between: (**a**) δ^13^C-CH_4_ and δ^13^C-DIC; (**b**) δ^2^H-H_2_O and δ^2^H-CH_4_; and (**c**) δ^18^O-H_2_O and δ^2^H-H_2_O for samples that contain CH_4_ > 10 μg/L between aquifers in the study area. For (**c**): GMWL = Global Meteoric Water Line; LMWL = Local Meteoric Water Line at Toowoomba^93^. For (**b**): long-dashed lines = range of combined hydrogen isotope effects for CO_2_-reduction as reported in Whiticar^3^, being δ^2^H-CH_4_ = δ^2^H-H_2_O–165‰ (±15‰); and short-dashed lines = range of combined hydrogen isotope effects for acetoclastic methanogenesis in sulfate-poor systems as reported in Waldron *et al.*^48^, being δ^2^H-CH_4_ = 0.675 × δ^2^H-H_2_O–284‰(±6‰). For (**b**), arrows represent the range of isotope effects where a combination of methanogenic pathways has potentially influenced isotopes as reported in Whiticar^3^. For (**a**), Alluvium–Free CH_4_^a^ = free gas samples taken from the well-head space of irrigation bores after extended pumping (up to 3 months) near Cecil Plains, as reported in Iverach *et al.*^21^.

In the gas reservoir, the δ^13^C-CH_4_ and δ^2^H-CH_4_ values ranged from −58‰ to −49‰, and −210‰ to −198‰, respectively, and correlate with positive δ^13^C-DIC values (+9‰ to +23‰) (Fig. 3a). The α_DIC-CH4_ and α_H2O-CH4_ of CSG production water are consistently around 1.07 and 1.2, respectively, and there is a positive relationship between the δ^13^C-CH_4_ and the δ^13^C-DIC (Fig. 3a,b). This, in combination with no other hydrocarbons above DL, is indicative of a biogenic CO_2_-reduction pathway in a closed system (limited CO_2_ pool). This is synonymous with gas trapping on geological structures in closed environments, such as anticlines and synclines. The predominance of biogenic CH_4_ in the coal measures in this basin is supported by a number of other studies, with the most recent work suggesting microbial CH_4_ in these reservoirs was generated since the late Pleistocene^26^,^27^,^28^,^29^.

In the shallow coal measures, the δ^13^C-CH_4_ and δ^2^H-CH_4_ ranged from 80‰ to −50‰, and −310‰ to −210‰, respectively. In the case of the alluvium, the data showed a similar range of −78‰ to −49‰, and −315‰ to −186‰, for δ^13^C-CH_4_ and δ^2^H-CH_4_, respectively. The range of δ^13^C-DIC values was also similar between the shallow coal measures and the alluvium: −15.9‰ to −3.5‰, and −15.3‰ to −6.6‰, respectively. The δ^13^C-CH_4_ and δ^2^H-CH_4_ and associated fractionation factors indicate CO_2_ reduction is the dominant pathway in the shallow coal measures, but variability in this data for the shallow coal measures and alluvium suggest there may be multiple production and/or consumption pathways influencing CH_4_ in these aquifers. The enriched δ^13^C-CH_4_ (−50‰) and highly depleted δ^2^H-CH_4_ (<310‰), and carbon and hydrogen fractionation factors of ~1.4 for a single shallow coal measure (P7) and alluvial sample (IND4) (Fig. 3a,b), are synonymous with acetoclastic methanogenesis^3^,^48^. These occur in isolation: under basalt sheetwash near Bowenville (shallow coal measures sample), and on the opposite side of the alluvium near Stratheden (alluvial sample) (see Figs 2 and S2). Acetoclastic methanogenesis has not been observed before in the Walloon Coal Measures in the Surat and Clarence-Moreton basins, although evidence of this pathway has been observed at basin margins in other areas^26^,^27^,^29^,^41^,^49^,^50^,^51^. In both cases these samples are found at relatively higher salinity and SO_4_ concentrations: for the shallow coal measures sample, Cl = ~1775 mg/L or 50 meq/L, and SO_4_ = 480 mg/L or 10 meq/L; for the alluvial sample, Cl = 5990 mg/L or 168 meq/L, and SO_4_ = 144 mg/L or 3 meq/L.

The only coal measure samples that contained hydrocarbons in addition to CH_4_ were found at a nested site at Cecil Plains: these samples (IND1 and IND3) contained small concentrations of ethene (70 and 25 μg/L). ethane (30 and 25 μg/L) and propene (24 and <10 μg/L) which suggests a mixed thermogenic/biogenic gas component. However, the depleted δ^13^C-CH_4_ of these samples (−71‰ and −65‰, respectively) as well as the α_DIC-CH4_ and α_H2O-CH4_ indicate biogenic CH_4_ is dominant at these sites.

### δ^18^O and δ^2^H in water

The CSG production water tends to be more isotopically depleted (ranging from −7.2‰ to −5.2‰, and −44.1‰ to −33.1‰, for δ^18^O and δ^2^H, respectively) than the shallow coal measures (ranging from −5.5‰ to −4.3‰, and −36.2‰ to −28.2‰, for δ^18^O and δ^2^H, respectively) and the alluvial water (ranging from −5.9‰ to −4.2‰, and −38.2‰ to −26.8‰, for δ^18^O and δ^2^H, respectively). This indicates that these deeper areas of the coal measures were recharged during cooler climates than the shallow coal measures and alluvium (Fig. 3c). These values are within the range previously reported for production water in the Surat Basin, which suggests recharge during the last glacial period in south east Queensland^27^,^28^. We found no evidence of a spatial relationship between the similarities in the stable isotopes of water from the alluvium and shallow coal measures samples and those from the gas reservoir (CSG production water): for example, the alluvial sample with depleted stable isotopes of water is found in a shallow well (18 m) located on the north eastern flank of the alluvium and is not related to the gas reservoir. Similarly, the most depleted shallow coal measures sample occurs in the ranges near a basalt outcrop. Some caution needs to be applied to interpretations of the stable isotope of water in CSG production water results because high rates of methanogenesis can influence the δ^2^H-H_2_O in closed systems^41^,^51^,^52^.

### Assessing potential migration from the CSG reservoir to the shallow coal measures

**Figure 4:**
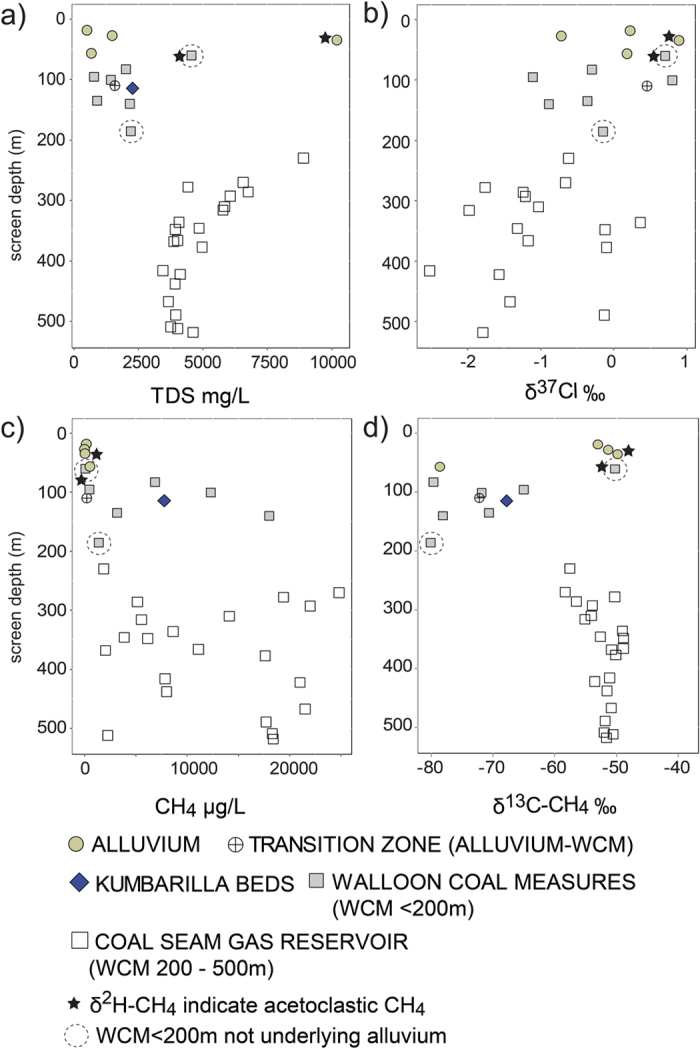
CSG groundwater and other groundwater samples that contain CH_4_ > 10 μg/L, showing: (**a**) TDS versus screen depth; (**b**) δ^37^Cl versus screen depth; (**c**) CH_4_ versus screen depth; and (**d**) δ^13^C-CH_4_ versus screen depth.

At the depth interface between the gas reservoir and the shallow coal measures there is a distinct shift in the relatively enriched δ^13^C-CH_4_ values of the gas reservoir samples, towards more depleted isotope values for samples from the shallow coal measures. Diffusion of CH_4_ may lead to lighter δ^13^C-CH_4_^12^ and a depletion of CH_4_ along a diffusion pathway^12^. Similarly diffusion of Cl would also lead to a distinct depletion of δ^13^Cl in combination with a decrease in TDS. However, for these data, an upward diffusion scenario from the CSG reservoir to shallower areas is not evidenced from the δ^13^Cl, TDS, CH_4_ or δ^13^C-CH_4_ data (Fig. 4a–d). This distinct change in the δ^13^C-CH_4_ values indicates that there is no evidence of leakage from the deeper gas reservoir to overlying shallow zones in the coal measures, either via diffusion or ebullition/advection. Therefore, the variability of the δ^13^C-CH_4_ in the shallow coal measures must be the result of changes in methanogenic pathways and/or consuming processes.

### The influence of SO_4_ on CH_4_ in the shallow coal measures

**Figure 5:**
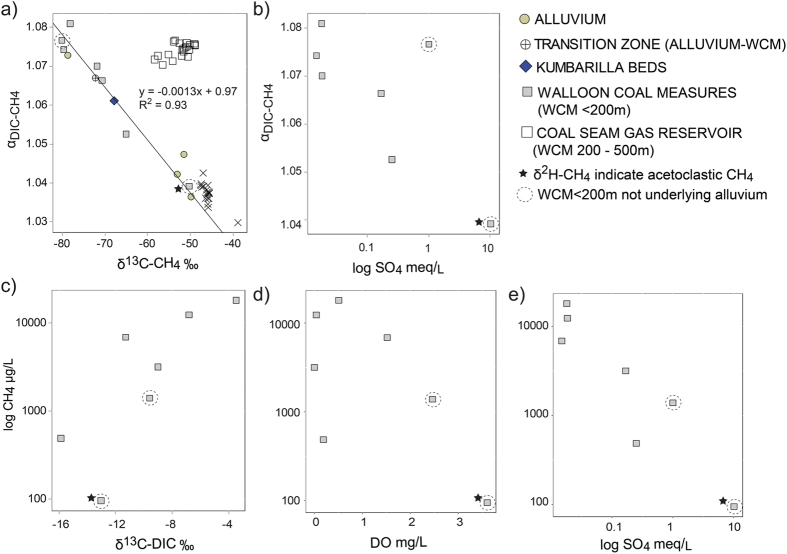
(**a**) δ^13^C-CH_4_ versus α_DIC-CH4_ values for all dissolved and free gas samples (regression line is for shallow coal measures (grey squares) only), as well as dissolved CH_4_ samples for shallow coal measures showing: (**b**) log SO_4_ meq/L versus α_DIC-CH4_ values; (**c**) δ^13^C-DIC versus log CH_4_ μg/L; (**d**) DO mg/L versus log CH_4_ μg/L; and (**e**) log SO_4_ meq/L versus log CH_4_ μg/L. For (**a**), Alluvium–Free CH_4_^a^ = free gas samples taken from the well-head space of irrigation bores after extended pumping (up to 3 months) near Cecil Plains, as reported in Iverach *et al.*^21^.

A decrease in the α_DIC-CH4_ as the δ^13^C-CH_4_ become more depleted in the shallow coal measures suggests influences of different methanogenic pathways (Fig. 5a). This is generally associated with a depletion of SO_4_ (Fig. 5b). The presence of SO_4_ can limit methanogenic activity, particularly for CO_2_ reducers, because SO_4_-reducing organisms are better at accessing both H_2_ and acetate^1^,^4^,^5^,^14^,^53^,^54^. In most cases the SO_4_ reducers maintain H_2_ levels below a threshold at which CO_2_ reducers can compete, resulting in complete inhibition of CO_2_ reduction. In contrast, acetoclastic methanogens, despite having a slower growth rate, can compete for acetate with SO_4_ reducers to the point where both organisms can co-exist^54^. Therefore, as SO_4_ depletes in the coal measures, we can expect changes in the methanogenic community, from one where CO_2_ reduction is inhibited by SO_4_ reducers and where some acetoclastic methanogenesis can occur, to one where CO_2_ reduction dominates over acetogens. This has implications for the δ^13^C-CH_4_ and could explain why the α_DIC-CH4_ changes as the δ^13^C-CH_4_ depletes (Fig. 5a). This hypothesis is supported by a decrease in SO_4_ concentrations as the CH_4_ increases (Fig. 5e). A positive relationship between CH_4_ concentrations and δ^13^C-DIC (Fig. 5c) demonstrates active methanogenesis and, where SO_4_ becomes depleted and CO_2_ reduction becomes dominant, higher CH_4_ concentrations indicate higher rates of methanogenesis via the CO_2_-reduction pathway. Data do not indicate an influence of DO concentrations on CH_4_ or associated isotopes (Fig. 5d), although methanogens can tolerate low concentrations of DO^55^. Spatially variable CH_4_ in the coal measures is supported by other recent studies which suggested variability in recharge as a possible influence^56^,^57^. In this study, variability of recharge may be contributing SO_4_ (either through discharge or pyrite dissolution) and DO, particularly in the shallower zones.

### Thermodynamic controls on CH_4_ in the shallow coal measures

In order to further explore the potential dynamism between CH_4_ production pathways, SO_4_ reduction, potential anaerobic oxidation of CH_4_ (AOM) and their influences on carbon and hydrogen isotopes at these large scales, we use a novel combination of thermodynamic information and changes in the activities of reactive species and isotope data expressed as isometric log ratios. A key aspect of this approach is understanding the behaviour of H_2_, which is a rate-limiting reactant for both CO_2_ reduction and SO_4_ reduction, while other reactants, such as HCO_3_ and SO_4_ may also provide favourable/unfavourable conditions for certain microbial pathways in coal seams^4^,^5^,^6^,^58^,^59^.

A sequential binary partition is used to calculate each isometric log ratio^60^. The sequential binary partition for each reactant shown in equations (6–8) (CO_2_ reduction, SO_4_ reduction and AOM, respectively) is shown in Tables 2, 3 and 4, respectively. The activity of H_2_O is ignored in relevant reactions, since it is always ~1. All ilr-coordinates are calculated using equation (1). In all isometric log ratio (ilr) calculations, the first ilr represents the compositional changes in the reaction pathway (products versus reactants). As a result, the first ilr (ilr.1) for each reaction pathway is similar to the reaction quotient (Q) used to calculate the change in Gibbs free energy. Using this approach, the principles of compositional data analysis and the law of mass balance holds, such that changes in the composition of species subsequently change the composition of the reactants and products. Where the reaction pathway is limited by the availability of one or more reactants, the ilr.1 is expected to follow a linear relationship with the changes in Gibbs free energy. The remaining ilr-coordinates partition the reactants into subcompositions, thus describing the availability of reactants for the reaction.

**Figure 6:**
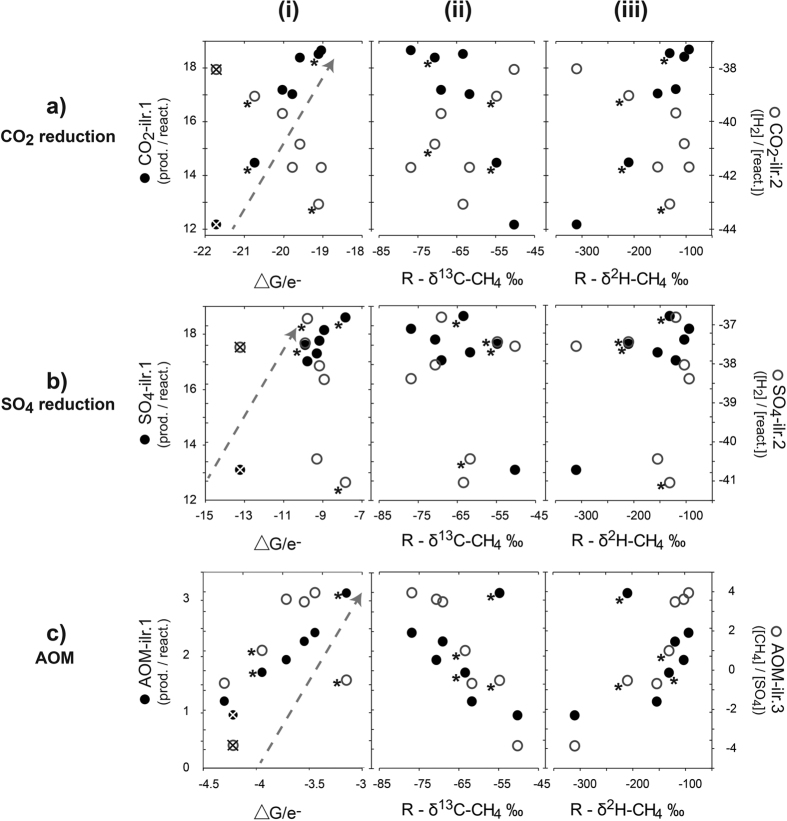
Microbial reaction pathways in the shallow coal measures (<200 m) for: (**a**) methanogenesis via CO_2_ reduction; (**b**) SO_4_ reduction; and (**c**) anaerobic oxidation of CH_4_ (AOM), showing comparison of isometric log ratios (ilr) derived from the partitioning of reactants and products (ilr.1–solid circles) and the partitioning of reactants (ilr.2–open circles) (see Tables 2, 3, 4 for respective SBPs) and: (i) changes in Gibbs free energy standardised to the number of electrons transferred for each reaction (8) (ΔG/e^−^); (ii) Rayleigh fractionation of δ^13^C-CH_4_; and (iii) Rayleigh fractionation of δ^2^H-CH_4_. Dashed arrows in (i) represent the direction in which the thermodynamic reaction proceeds (approaches equilibrium). Under acetoclastic methanogenesis the δ^13^C-CH_4_ can be relatively enriched, yet should become more depleted as CO_2_ reduction proceeds. In the same context, the δ^2^H-CH_4_ is highly depleted under acetoclastic methanogenesis, yet more enriched under CO_2_ reduction. As a result, a reciprocal response between carbon and hydrogen isotopes is expected as the reaction pathway changes: therefore, the R-δ^13^C-CH_4_ is defined as R = R_i_ *f* ^ (1−α)^, while R-δ^2^H-CH_4_ is defined as R = R_i_ *f* ^ (α−1)^, where *f* = 0 = min CH_4_. The acetoclastic sample is marked with a cross in (i). *Samples containing ethene (70 and 25 μg/L), ethane (30 and 25 μg/L) and propene (24 and 0 μg/L).

For the CO_2_ reduction pathway scenario (Fig. 6a(i–iii)), a decrease of H_2_ relative to other reactants (H^+^ and HCO_3_^−^) (CO_2_-ilr.2) occurs as the reaction pathway proceeds (ΔG/e^−^ become less negative). This can be interpreted as the consumption of H_2_ as methanogenesis proceeds and as SO_4_ is depleted. The inverse relationship with the CO_2_-ilr.1 (reactants vs products) shows that the availability of H_2_ in higher SO_4_ environments is limiting CO_2_ reduction pathways. A depletion in the relative R-δ^13^C-CH_4_ and enrichment of R-δ^2^H-CH_4_ isotope along this pathway support a shift from acetoclastic methanogenesis in higher SO_4_ environments where competition from SO_4_ reducers is higher to one where CO_2_ reduction becomes dominant in lower SO_4_ environments. In addition to low SO_4_ concentrations, low H_2_ and low HCO_3_ concentrations can also create more favourable conditions for CO_2_ reducers^59^.

For the SO_4_ reduction pathway (Fig. 6b(i–iii)), poor relationships between all SO_4_-ilr.2, ΔG/e^−^ and isotopic responses was observed. This indicates different controls on the SO_4_ reduction pathway: it does not appear to be limited by the availability of reactants, including H_2_ and, with the exception of the obvious acetoclastic sample, does not appear to accompany a distinct carbon or hydrogen isotopic response.

Increases (less negative) in the ΔG/e^−^ for the AOM pathway are accompanied by an increase in the relative concentration of CH_4_ to SO_4_ (AOM-ilr.3), which shows that as AOM proceeds, the system moves towards one where the CH_4_/SO_4_ ratio increases (Fig. 6c)(i–iii). This indicates that, as the AOM reaction approaches thermodynamic equilibrium, the amount of SO_4_ available for the reaction decreases, yet CH_4_ must continue to be produced. The availability of SO_4_ appears to be a limiting reactant for the AOM pathway. These relationships are accompanied by a relative depletion of R-δ^13^C-CH_4_ and enrichment of R-δ^2^H-CH_4_ values as CH_4_ concentrations increase. Any CH_4_ oxidation in higher SO_4_ environments, as well as the slow growth rate of acetoclastic methanogens, is likely to contribute to the relatively lower CH_4_ concentrations in higher SO_4_ environments. In some cases AOM can occur in tandem with methanogenesis^61^. However, due to generally low S_2_^−^ and HS^−^ being <DL for all samples, we do not expect the influence of AOM to be significant when compared with the influence of SO_4_ and shifts from acetoclastic methanogenesis to CO_2_-reduction. At an isolated site underlying a basalt outcrop (P19), NO_3_ concentrations were slightly above DL (0.01 mg/L) at 0.02 mg/L (3.23e-04 meq/L), but the highly depleted δ^13^C-CH_4_ (−80‰) at this site does not suggest oxidation via denitrification is occurring.

### Assessing potential migration of CH_4_ from the shallow coal measures to the alluvium

#### Nested sites

Three (n = 3) nested sites that include wells in the underlying coal measure and overlying alluvium wells were sampled: (1) Cecil Plains; (2) Stratheden; and (3) Dalby (see Figure S2). At all sites CH_4_ was observed in the underlying shallow coal measures, or the Kumbarilla Beds, but no CH_4_ was found in the alluvial wells.

At the Stratheden nested well site (IND4, IND5, IND6), water levels are similar, indicating there is not a significant pressure gradient to induce groundwater flow, and the absence of CH_4_ in the intermediate well does not suggest upward CH_4_ at this site. The δ^13^C-CH_4_ of the alluvial CH_4_ at this site is more enriched (−50‰) when compared with the deeper Kumbarilla CH_4_ sample (−68‰): the highly depleted δ^2^H-CH_4_ (−315‰) of the alluvial sample at this nested site (IND4) indicates acetoclastic methanogenesis^3^,^48^.

At the Cecil Plains nested site (P20, IND1, IND2, IND3) the sample with peak DOC (7 mg/L) (IND2) occurred in the alluvial-coal measure transition zone (85 m, ~7 m below the alluvial basement), but the DOC of the overlying alluvial sample was significantly lower (0.3 mg/L). On the same note, the CH_4_ samples from the two coal measures samples at this site is accompanied by small concentrations of ethene (70 and 25 μg/L). ethane (30 and 25 μg/L) and propene (24 and <10 μg/L); yet we found no other hydrocarbons in the alluvial sample (DL for all hydrocarbons = 10 μg/L). Similarly, at the Dalby nested site (GM1390, GM1074) no CH_4_ was found in the alluvial well (see Figure S2 for details of nested sites). We conclude that no CH_4_ migration from the underlying coal measures into the alluvium is occurring at these sites.

#### Dissolved CH_4_ in the alluvium

**Figure 7:**
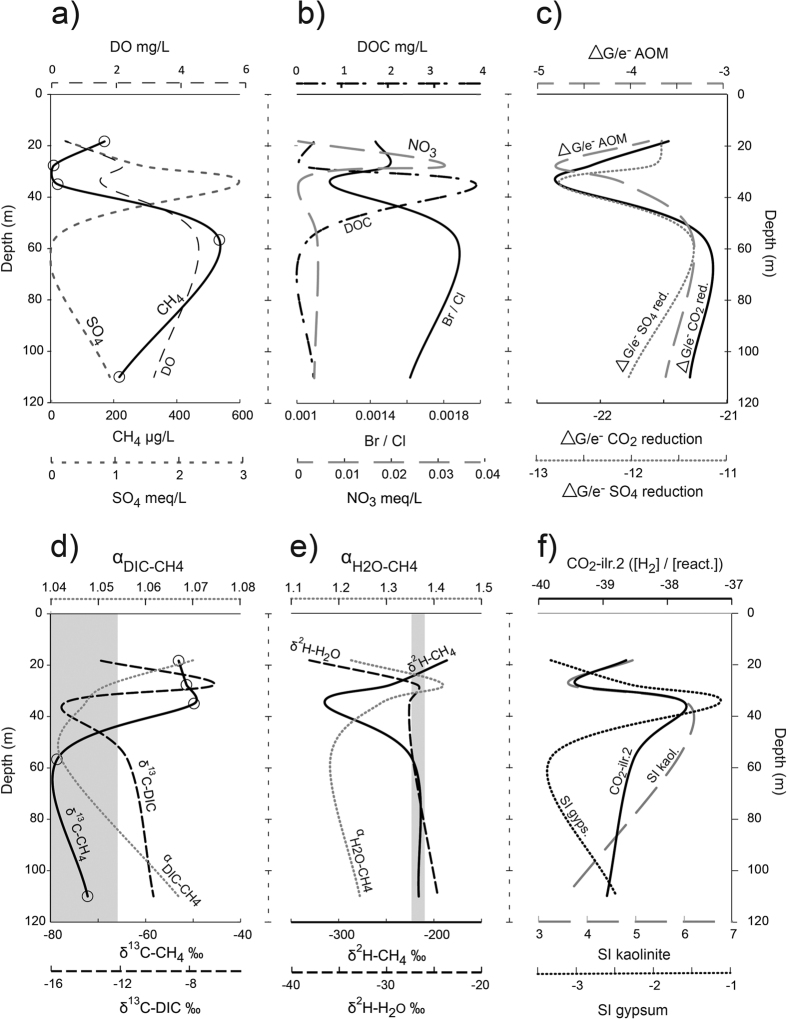
Comparison of various parameters in the alluvial depth profile for sites with dissolved CH_4_ > DL (10 μg/L), showing: (**a**) CH_4_ (μg/L), SO_4_ meq/L and DO mg/L; (**b**) Br/Cl ratio, NO_3_ mg/L and DOC mg/L; (**c**) ΔG/e^−^ for CO_2_ reduction, SO_4_ reduction and AOM pathways; (**d**) the carbon isotopes of CH_4_ and DIC phases and their respective fraction factors; (**e**) the hydrogen isotopes of CH_4_ and H_2_O phases and their respective fractionation factors; and (**f**) CO_2_.ilr-2 (the ilr of [H_2_]/[reactants of CO_2_ reduction/SO_4_ reduction pathways] (see Tables 2 and 3)), and the saturation of indices of kaolinite and gypsum. Circles in (**a**,**d**) represent the sample point depth: this corresponds to the sample point for all parameters in all plots. Shaded areas in (**d**,**e**) represent the ranges of δ^13^C-CH_4_, δ^2^H-CH_4_, respectively for the coal measures that directly underlie the alluvium.

Results show that the CH_4_ in the shallow coal measures that directly underlie the alluvium are depleted in δ^13^C-CH_4_ (−80‰ to −65‰), and have δ^2^H-CH_4_ between −222‰ and −209‰. Fractionation factors and thermodynamic results indicate CH_4_ in the shallow coal measures is generated predominantly via the CO_2_ reduction pathway, with SO_4_ concentrations being a major control. Subsequently, the assessment of potential migration of CH_4_ from the coal measures to the alluvium must consider this CH_4_ of the shallow (underlying) coal measures as the appropriate end member. The lack of evidence of CH_4_ leakage from the gas reservoir to the shallow coal measures, and the abrupt shift from enriched δ^13^C-CH_4_ (−58‰ to −49‰) of the gas reservoir to the depleted δ^13^C-CH_4_ of the shallow coal measures indicate that the migration of CH_4_ from the gas reservoir to the alluvium at these sites is not a plausible scenario based on these data. Variability in the TDS and CH_4_ concentrations, as well as the δ^37^Cl (Fig. 4), and similar ranges of δ^13^C-CH_4_ and δ^2^H-CH_4_ between the underlying coal measures and deep alluvial samples (Fig. 7d,e) also do not suggest diffusion of CH_4_ from the underlying coal measures to the alluvium.

No relationship between depth and CH_4_ concentration in the alluvium was observed at sites sampled in this study, with the highest concentrations occurring at ~60 m (Fig. 7a). Thermodynamic conditions in the alluvium are suitable for all reaction pathways to occur (Fig. 7c); however, the variability of CH_4_ concentration in the alluvium is related to the inverse of SO_4_ concentration (Fig. 7a), demonstrating the influence of SO_4_ reduction on methanogenic activity. High concentrations of SO_4_ accompany high TDS (salinity) and large decreases in the Br/Cl ratio (Fig. 7a,b). This shows that different controls on salinity influence these high SO_4_ concentrations. The relatively consistent δ^2^H-H_2_O at maximum salinity shows that the CH_4_ and related hydrochemical conditions are not necessarily related to different sources of water or simple evaporation processes, and are more likely to reflect the accumulation of salts, including gypsum, or transpiration at a less permeable zone. Interestingly, the high-SO_4_ zone coincides with depleted δ^2^H-CH_4_ values (-315‰) that indicate acetoclastic methanogenesis (Fig. 7e). This explains the more enriched δ^13^C-CH_4_ for this sample (IND4) and it is a similar scenario to that which we observed in the coal measures.

δ^13^C-CH_4_ and δ^2^H-CH_4_ values for two deep alluvial samples (GM1193 and GM0057: 110 m and 57 m, respectively) are within a similar range to that of the underlying coal measures (Fig. 7d,e). The deepest site (GM1193) is at/near the alluvial-coal measure transition zone. As stated previously, there are small coal fragments in the sandy alluvial deposits at this site, which could provide a methanogenic substrate. Furthermore, the δ^18^O of this sample is the most enriched of the CH_4_ data set and does not suggest a coal measure source (Fig. 3c). Similarly, the δ^13^C-CH_4_ and δ^2^H-CH_4_, as well as the α_DIC-CH4_ and α_H2O-CH4_, are also consistent with *in situ* CO_2_ reduction at the deepest site (Fig. 7d,e), and do not suggest an oxidation pathway or CH_4_ sourced from another area/zone.

Where peak CH_4_ concentrations occur (~57 m: GM0057), the α_DIC-CH4_ values are as low as ~1.04 (Fig. 7d), but the depleted δ^13^C-CH_4_ and enriched δ^2^H-CH_4_ do not support acetoclastic methanogenesis at this site. While α_DIC-CH4_ values of ~1.07 are typical of CO_2_ reduction, a fractionation factor of 1.04 is still within the range observed for CO_2_ reduction^5^,^62^,^63^,^64^. These fractionation factors can change between sites and as a function of *in situ* conditions^5^,^6^. Low ΔG/e^−^ values at this site may also suggest some AOM has occurred (Fig. 7c). Alternatively the acetate- and H_2_-dependent methanogenesis may also occur concurrently during acetate fermentation in some cases^65^,^66^. Well GM0057 is located near the river and may also receive some river recharge. This well, and well GM1193, occur in a sandy area of the aquifer where pumping rates and recharge are likely to be relatively higher than areas around Dalby; this could explain slightly higher DO concentrations (Fig. 7a). Methanogenesis may also persist in the presence of low DO concentrations^55^, and mixing of slightly oxygenated water (river recharge) and the dissolution of carbonates may explain the relatively lower α_DIC-CH4_ values at GM0057.

In the shallow alluvial zones, fluxes in the type and rate of methanogenesis could be influenced by wetting and drying periods that result in dissolution or precipitation of minerals such as gypsum (Fig. 7f). In addition, clay mineral content has also been shown to influence CH_4_ concentrations, with high clay content capable of trapping CH_4_^67^. Furthermore, some clays (e.g. kaolinite) preserve organic matter better than others^68^. For samples analysed in this study, kaolinite saturation indices are highest in the high-SO_4_ zone where acetoclastic methanogenesis dominates (Fig. 7f), which also accompanies a peak in DOC concentrations (Fig. 7b). The presence of kaolinite clay lenses in shallow areas may have a dual effect on methanogenic activity by preventing flushing and promoting salinization that result in higher SO_4_, as well as the preservation of some organic matter that allows fermentation processes to persist. The proportion of [H_2_] to other reactants ([HCO_3_] and [H^+^]) (CO_2_.ilr-2) in the more saline/high SO_4_ zone increases (ratio of H_2_ to HCO_3_ increases), despite CH_4_ being low (IND4): this indicates a fermentation process by SO_4_ reducers and acetate-dependent methanogens.

While AOM is thermodynamically favourable, slightly more depleted δ^13^C-CH_4_ and α_DIC-CH4_ values ~1.07 in the shallower zones do not suggest significant AOM is occurring (Fig. 7d,e). Mixing processes in the shallow alluvium may create scenarios where water with CH_4_ is mixing with water with higher concentrations of redox species. For one sample (GM1076), a small increase in the NO_3_ concentration is evident (Fig. 7b) and the oxidation of small amounts of CH_4_ via denitrifying bacteria cannot be completely ruled out^69^,^70^, although we found no NO_2_ above DL at any sites, and fractionation factors support a CO_2_ reduction pathway. Substrate depletion may also explain enriched δ^13^C-CH_4_ in these shallow zones^3^. Some caution should be applied when drawing conclusions for the shallowest alluvial samples (GM1073 and GM1076) because the measured δ^13^C-CH_4_ was at or near the limit of quantification (0.8 nanomoles) for the analytical method (this is not the case for δ^2^H-CH_4_). However, we note that deeper alluvial wells in this area of the alluvium, which is adjacent to the deep gas reservoir, did not contain CH_4_ above DL (10 μg/L). As a result, CH_4_ migration from the underlying coal measures in this area does not seem likely at these sites.

#### A conceptual model of CH_4_ within and between aquifers

**Figure 8:**
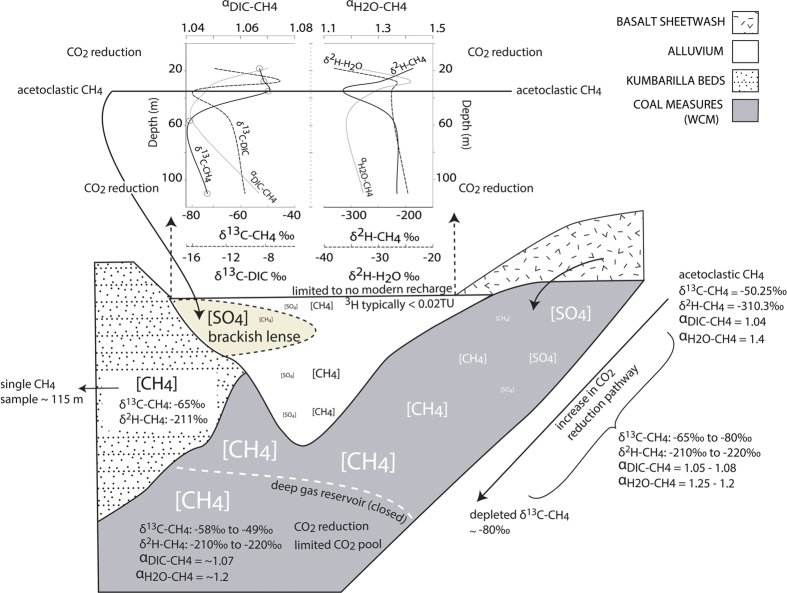
Conceptual model of the behaviour of carbon and hydrogen isotopes in CH_4_ and respective DIC and water phases in the alluvium and the underlying coal measures. The two graphs at the top are related to the alluvium only. The δ^13^C-CH_4_ in the deep gas reservoir (200–500 m) is typically enriched, relative to the δ^13^C-CH_4_ in the shallower (<200 m) parts of the coal measures. In both the alluvium and the shallow coal measures CH_4_ concentrations are controlled by SO_4_ concentrations, with acetoclastic methanogenesis detected in shallow high-SO_4_ zones. CO_2_ reduction is the dominant pathway in both the coal measures and the alluvium. In the shallow coal measures anaerobic oxidation of CH_4_ and acetoclastic methanogenesis maintain depleted (~-50‰) δ^13^C-CH_4_, but, as SO_4_ depletes with depth, CO_2_ reduction becomes the dominant pathway and the δ^13^C-CH_4_ subsequently depletes (~−80‰). A single CH_4_ sample was detected in the Kumbarilla Beds, but there are insufficient wells in this formation to accurately assess controls on CH_4_; however, a shallower well in Kumbarilla beds at the same site contained no CH_4._

A conceptual model (Fig. 8) summarises the major controls on CH_4_ within and between aquifers, including:

1. Closed system conditions leading to enriched δ^13^C-CH_4_ and positive δ^13^C-DIC in the deep gas reservoir (200–500 m); and
2. The presence SO_4_ concentrations and its influence on methanogenic pathways, including shifts from the acetoclastic pathways in shallow, brackish- and high SO_4_- zones to a dominance of CO_2_ reduction in deeper, low SO_4_ zones, in both the shallow coal measures and the alluvium.

The inverse relationships between CH_4_ and SO_4_, and associated isotopic responses and thermodynamic conditions, in the shallow coal measures and alluvium are consistent with *in situ* CH_4_ production in other freshwater and brackish environments^3^,^4^,^5^,^6^,^71^. This, combined with results at nested sites and an absence of CH_4_ > DL (10 μg/L) in the alluvium, does not suggest large-scale migration of CH_4_ from the underlying coal measures is occurring.

Due to the complexity of methanogenesis and methantrophy in the subsurface, different pathways and sources can result in similar δ^13^C-CH_4_ values (see Figure S1), and CH_4_ and δ^13^C-CH_4_ are not likely to be spatially consistent. Enriched δ^13^C-CH_4_ from CSG production water can pertain to gas trapping scenarios at discrete locations^3^,^26^,^72^ and these values are not necessarily representative of CH_4_ in the entire aquifer. For future studies that are concerned with understanding CH_4_ behaviour in the subsurface over large areas and/or associated with CSG, we propose the following parameters as a minimum standard for data collection: δ^13^C-CH_4_ and δ^2^H-CH_4_, δ^13^C-DIC, major ions, pH and SO_4_ and S_2_^−^ (other redox species, such as Fe and NO_3_, may also have some value). Researchers are encouraged to prepare comprehensive data sets of a range of parameters that allow conceptual models of the extent, and influences on, CH_4_ within and between aquifers to be described and built upon over time. The conceptual model outlined here (Fig. 8) provides a basis for doing this in this catchment. More sampling to identify the presence of methanogenic consortia (culturing studies) within and between aquifers, including the extent of acetoclastic methanogens, would build on the information collected in this study.

### Comparisons with free gas measurements from alluvial wells

The results presented here are not in agreement with another study in the Cecil Plains area which used δ^13^C-CH_4_ of free CH_4_ taken from multi-screened irrigation wells during pumping to infer CH_4_ leakage from the coal measures to the alluvium at four sites^21^. That study proposed the following be met to infer CH_4_ migration from the underlying coal measures:

1. DOC > DL, and ^3^H < QL (0.04 TU), where QL is quantification limit (this relationship inferred a potential source of coal measure groundwater/CH_4_); and
2. Samples must sit on a mixing line between 1/CH_4_ and δ^13^C-CH_4_, with a *y*-axis intercept with a δ^13^C-CH_4_ value of −55.9‰.

That study assumed that the δ^13^C-CH_4_ value of −55.9‰ used in their mixing line is representative of the CH_4_ in entire coal measure aquifer, and that there are only two sources of DOC: river recharge or discharge from the coal measures. A δ^13^C-CH_4_ value of −50.8‰, based on a single atmospheric measurement downwind of a CSG production water storage pond, was also used as a reference (end-member) value for the coal measures aquifer. However, that study did not take any samples from the coal measures, either underlying the alluvium or in other areas, for reference.

#### Relationships between DOC and CH_4_

We found DOC in the alluvium (and the coal measures) to be relatively low, yet within a consistent range, regardless of distance from the river or tritium activity. Advanced analytical techniques are required to confidently detect tritium at low TU. We used a highly sensitive tritium analytical technique (DL = 0.02 TU)^73^, yet only found tritium >DL at two alluvial wells (GM1076 and GM1338). Iverach *et al.*^21^ suggested that, where tritium was below QL, yet DOC is present, a source of DOC, in addition to river recharge, must be present. These authors proposed that “upwards migration of CH_4_ from the coal measures would be the most likely source” of DOC in the alluvium at these sites; however, CH_4_ is not part of the DOC pool.

Other studies have indicated that a diffuse recharge component over the alluvium is possible in this catchment^23^,^74^,^75^, which may contribute to the DOC pool in the alluvium. In addition, DOC can diffuse through clays and/or be preserved by some clays such as kaolinite, and DOC can also be generated *in situ* in the subsurface from sedimentary sources^39^,^68^,^76^. Therefore, DOC and CH_4_ are likely to be associated with different sources and transport mechanisms. Furthermore, CO_2_ reduction is the dominant methanogenic pathway in the coal measure and alluvial aquifers, and this pathway does not rely on DOC as the energy source, rather it uses H_2_^2^ (equation 6). We suggest that a more comprehensive research approach is needed to better understand relationships between DOC, age tracers, such as tritium, and methanogenesis within and between aquifers before combinations of these parameters can be used to confidently validate aquifer connectivity, particularly when working at large scales.

#### Describing the coal measure CH_4_ end member

The enriched δ^13^C-CH_4_ value (55.9‰) estimated for the regression line used by Iverach *et al.*^21^ to infer CH_4_ leakage from the coal measures to the alluvium is within the range of the δ^13^C-CH_4_ observed for the deeper gas reservoir (>200 m) sampled in our study (−58‰ to −49‰), and other gas reservoirs in the Surat Basin^27^,^28^. However, it contrasts with the depleted δ^13^C-CH_4_ (−80‰ to −65‰) that we observed for the shallow (<200 m) coal measures that underlie the alluvium. In this study area the CSG reservoir occurs in deeper zones (>200 m) of the coal measures where gas trapping occurs on the north-western flank of the alluvium (Figure S2). These conditions produce enriched δ^13^C-CH_4_ and high, positive δ^13^C-DIC (Fig. 4a) in the gas reservoir that do not occur in the shallower coal measures directly under the alluvium. High and positive δ^13^C-DIC can be an excellent indicator of CSG production water migration^77^; yet highly enriched/positive δ^13^C-DIC values were not found in the shallow coal measures or the alluvium, either in this study or by Iverach *et al.*^21^. The only enriched δ^13^C-CH_4_ (~50‰) we observed for the shallow coal measures was an isolated acetoclastic CH_4_ sample (P7) that underlies basalt sheetwash (see Figure S2).

The majority of δ^13^C-CH_4_ of free CH_4_ measured in Iverach *et al.*^21^ are similar to background CH_4_ concentrations observed in that study and for ambient air in the southern hemisphere observed in other studies^78^,^79^,^80^. It is possible that most of the CH_4_ analysed in Iverach *et al.*^21^ were composed of atmospheric CH_4_. Additional sampling (preferably using low flow techniques) to measure the degassing rate/potential from alluvial groundwater would also assist in more accurately describing the proportion of atmospheric versus degassed CH_4_ in the well-head spaces measured by Iverach *et al.*^21^. Where mixing with atmospheric and subsurface-derived CH_4_ is shown to occur, simple mixing lines may be inadequate to understand mixing of the three theoretical end members that should be considered under these potential inter-aquifer CH_4_ migration scenarios: i.e. (1) atmospheric CH_4_; (2) alluvial-derived CH_4_; (3) CH_4_ that has migrated from other aquifers.

Hydrogen isotope analyses can reduce uncertainties associated with interpretations that are based solely on δ^13^C-CH_4_ values, as presented in Iverach *et al.*^21^. Atmospheric CH_4_ tend to be much more enriched in δ^2^H-CH_4_ values (−82‰) compared to biogenic CH_4_ (−160‰ to >−400‰)^3^,^48^,^80^. In addition, CH_4_ oxidation could partly explained the enriched δ^13^C-CH_4_ values (−47.4‰ to −38.8‰) measured in Iverach *et al.*^21^. Hydrogen isotope data can also provide information about the influence of oxidation, as well as different production pathways, on the isotopic composition of CH_4_^3^,^48^.

## Conclusions

Using a comprehensive data set (dissolved CH_4_, δ^13^C-CH_4_, δ^2^H-CH_4_, δ^13^C-DIC, δ^37^Cl, δ^2^H-H_2_O, δ^y^O-H_2_O, Na, K, Ca, Mg, HCO_3_, Cl, Br, SO_4_, NO_3_ and DO) this study described hydrochemical/thermodynamic controls on CH_4_ in a deep coal seam gas (CSG) reservoir (200–500 m), shallower areas of the same coal-bearing formation (the Walloon Coal Measures) (<200 m) and the overlying Condamine River alluvium (Surat/Clarence Moreton basins), eastern Australia. Enriched δ^13^C-CH_4_ (−58‰ to −49‰) and positive δ^13^C-DIC (+9‰ to +23‰) in the deep gas reservoir are synonymous with biogenic methanogenesis in closed-system conditions and gas trapping on geological structures. Evidence of leakage from the deep gas reservoir, either via diffusion or ebullution/advection, was not observed, with δ^13^C-CH_4_ of the shallow coal measures underlying the alluvium being depleted (−80‰ to −65‰). Importantly, this study demonstrates that, when evaluating potential CH_4_ migration associated with CSG, the enriched δ^13^C-CH_4_ of CSG CH_4_ is not necessarily the appropriate isotopic end member because the δ^13^C-CH_4_ in areas outside of gas reservoirs, yet within the same sedimentary formation, can be distinctly different due to different hydrogeological and microbial conditions. We found the δ^13^C-CH_4_ of the alluvium falls within a similar range to that of the shallow coal measures.

Using a novel application of isometric log ratios, this study demonstrated a simple method of providing insight into the microbial controls on δ^13^C-CH_4_ and δ^2^H-CH_4_ isotopes in the subsurface. The major controls on CH_4_ in the shallow coal measures and the alluvium were found to be: (a) the presence of SO_4_ and associated competition between SO_4_ reducers and CO_2_ reducers; and (b) shifts from acetoclastic methanogenesis in shallow, high-SO_4_ zones to the dominance of the CO_2_ reduction pathway in low-SO_4_ environments. AOM was found to be thermodynamically favourable but there was no evidence of large-scale, significant AOM in the shallow coal measures or the alluvium. Overall, this study did not find conclusive evidence of CH_4_ migration to the alluvium from the underlying (shallower <200 m) coal measures, but results do suggest small concentrations of CH_4_ are likely to be generated *in situ* in the alluvial aquifer at these sites. This study provides a comprehensive assessment using novel samples. More research and sampling in the area, including culturing studies of methanogenic consortia, will improve our understanding of the nature and extent of CH_4_ within and between aquifers.

## Methods

### Sample collection

Samples were collected from 61 wells, including: (a) monitoring wells and stock and domestic wells where there was sufficient space to lower a bladder pump^81^; (b) irrigation/domestic wells that already contained submerged electric pumps; and (c) production water wells. Where a bladder pump could be lowered into a well, the low-flow sampling technique was applied using a flow-through cell^81^. For government monitoring wells, where monitoring wells had multiple screens, the bladder pump was placed at the interval of the lowest screen. Infrastructure at irrigation/domestic wells prevented well dipping: in these cases a conservative water level estimate of ~75% of well depth was applied to consider a purging volume. Sampling coincided with landholders pumping schedules and, as a result, the majority of irrigation/domestic wells had been purged by at least 3 x well volume upon arrival on site. In all cases (low-flow sampling and irrigation/domestic-well sampling) sampling was only initiated after hydrochemical parameters (pH, temperature, specific conductance and DO) were stabilised^81^,^82^. Due to limited infrastructure, two operating windmills were sampled in recharge areas on the ranges (P9 and P16): in these cases sampling was conducted after a minimum of 3 days of consistent moderate-strong wind (consistent pumping to purge the well) and after stabilisation of hydrochemical parameters (pH, temperature, specific conductance and DO) were confirmed^83^. Coal seam gas production wells (deep gas reservoir) are constantly pumping and were considered adequately purged upon arrival on-site. Production water from CSG wells was sampled at an outlet of the extraction well prior to the gas-water separator.

Samples were taken from alluvium (n = 23), the Kumbarilla Beds (n = 3), the shallow Walloon Coal Measures (WCM) (<200 m) (n = 14) and from the deeper (200–500 m) gas reservoir in the coal measures (n = 21). Two of the alluvial samples taken were at the alluvial-WCM interface (see section 2). Wells were selected based on drill log information and previous interpretations of hydrogeology in the catchment^23^.

Samples for dissolved CH_4_ were collected in glass vials with rubber septums and no headspace (preserved with sulfuric acid). Samples for δ^13^C-CH_4_, δ^2^H-CH_4_ and δ^13^C-DIC were filtered through 0.2 micron filters and collected in 12ml entertainer vials with rubber septums and no headspace. Samples for cations, dissolved metals, δ^37^Cl and Br were filtered through high capacity in-line 0.45 μm polyethersulphone filters. Cation and dissolved metal samples were preserved in the field using HNO_3_ to pH <2. Samples for ^3^H and anions, NO_3_/NO_2_, S_2_^−^ and unionized HS were collected in unfiltered 1L Nalgene bottles, and HDPE bottles respectively (APHA Table 1060:1). NO_3_ and NO_2_ samples were preserved in the field using H_2_SO_4_ to pH <2. S_2_^−^ and unionized HS samples were preserved in the field with Zn acetate/NaOH. Bottles not containing a preservative were rinsed three times with sample water prior to collecting a sample. All samples, with the exception of ^3^H, were placed immediately on ice and stored on ice in the field, then in dark cold rooms (<4 °C) until analysis.

### Sample analysis

Samples were analysed for pH, DO, specific conductivity (conductivity) and temperature using a YSI physico-chemical meter in the field (YSI Professional Plus). Water samples were taken and analysed in the laboratory for major and minor ions (APHA 2320; APHA 3125B) including SO_4_ (APHA 4500 SO4-E–laboratory 0.45 μm filtered), and Br (APHA 4110 B) as well as unionised HS (APHA 4500-S2-H) and S_2_^−^ (APHA 4500-S^2^–D), NO_3_ and NO_2_ (APHA VCl reduction 4500 NO_3_^−^ + NO_2_^−^B), Fe and Mn (APHA 3125B ORP/ICP/MS Octopole Reaction Cell) and dissolved CH_4_ concentrations (including C1–C4 gases, DL = 10 μg/L: ALS EP033) at the Australian Laboratory Services laboratory, Brisbane, Queensland, and at Queensland Health Scientific and Forensics services laboratory (Br). Bicarbonate values are reported as bicarbonate alkalinity. All major and minor ions, and dissolved C1-C4 hydrocarbons were analysed within ~7 days of collection in the field.

δ^2^H and δ^18^O were measured using a Los Gatos Research Water Isotope Analyzer (QUT, Institute for Future Environments), with replicate analyses indicating an analytical error of 0.04‰ to 0.45‰, and 0.001‰ to 0.7‰, respectively.

δ^13^C-CH_4_ and δ^2^H-CH_4_ were measured using a ThermoScientific PreCon concentration system interfaced to a ThermoScientific Delta V Plus isotope ratio mass spectrometer at the UC Davis Stable Isotope Facility. Standard error of analysed samples was ~0.1‰, for δ^13^C-CH_4_ and ranged from 0.9–1.7‰ for δ^2^H-CH_4_, and limit of quantification = 0.8 and 2 nanomoles respectively. δ^13^C-DIC were also measured at the UC Davis Stable Isotope Facility using a GasBench II system interfaced to a Delta V Plus isotope ratio mass spectrometer. Standard error of δ^13^C-DIC ranged from 0.04–0.09‰: limit of quantification = 150 nanomoles.

δ^37^Cl were measured using a stable isotope ratio mass spectrometer at Isotope Tracer Technologies in Waterloo, Canada. Standard error ranged from 0.03–0.16‰.

The DOC analyses were performed on a Dohrmann DC-190 Total Carbon Analyzer at the Earth and Environmental Sciences at the University of Waterloo, Canada. DOC storage times (0.45 μm filtered, dark storage <4 °C) prior to analysis ranged from 260–620 days. Data Tables S1 and S4 report the measured and corrected DOC values, as per Peacock *et al.*^84^: these values are broadly similar with modelled loss of DOC being minimal due to low DOC concentrations.

Tritium (^3^H) samples were vacuum distilled and electrolytically enriched prior to liquid scintillation spectrometry analysis by Quantulus ultra-low-level counters at GNS, New Zealand^73^. The sensitivity is now further increased to a lower DL of 0.02 TU (two sigma criterion) via tritium enrichment by a factor of 95, and reproducibility of tritium enrichment of 1% is achieved via deuterium-calibration for every sample. The precision (1σ) is ~1.8% at 2 TU.

### Data preparation

All major ion data was above DL (DL) of 1 mg/L, with the exception of SO_4_ (n = 24). All S_2_^−^ measurements with the exception of 1 coal measures sample (well IND3; S_2_ = 0.5 mg/L, or ~0.008 meq/L) were below DL (DL = 0.1 mg/L, or 1.56e-03 meq/L). Low SO_4_ and S_2_^−^ concentrations are expected for reduced environments where methanogenesis occurs. Values below DL were imputed using the R package zCompositions via the log ratio Data Augmentation function (lrDA): this function is based on the log-ratio Markov Chain Monte Carlo MC Data Augmentation (DA) algorithm^85^.

### Deriving isometric log ratios

The isometric log ratio (ilr) uses a sequential binary partition (Table 5) to describe orthonormal bases to which correspond D-1 Cartesian coordinates (ilr-coordinates): these orthonormal coordinates, called balances, are orthogonal^86^. This technique removes potentially spurious correlation caused by scaling and allows the ratios of parts and subparts to be elucidated, even when the concentrations of different parts are relatively small compared to other parts. Here we use ilrs to investigate subcompositional relationships between reactants and products in a number of thermodynamic reaction pathways (CO_2_-reducing methanogenesis, SO_4_ reduction and anaerobic oxidation of CH_4_ (AOM)). These relationships are compared to isotope fractionation of the δ^13^C-CH_4_ and δ^2^H-CH_4_ under various conditions. This approach allows subcompositional behaviour of dissolved constituents to be compared to isotopic responses, in order to demonstrate the relationship between methanogenic activity/pathways, thermodynamic conditions and hydrochemistry.

Each partition divides the composition into separate parts (*x*_*i*_ and *x*_*j*_). For thermodynamic reaction pathways, we use the first partition to separate the activity of the element (represented by [element]) from reactants and products in each reaction, with the following partitions separating the reactants. Once a sequential binary partition is described, the i-th ilr balance is computed as


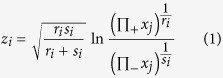


where *r*_*i*_ and *s*_*i*_ are the number of parts coded in the sequential binary partition as +1 and −1, respectively^86^. Isometric log ratios were calculated using CodaPak 2.10^87^.

### Describing isotope partitioning (fractionation factors)

The partition of isotopes between phases, e.g. between the dissolved inorganic carbon (DIC) and CH_4_ phase, can be described in a number of ways. For simplicity and reproducibility, here we define the isotope partition as the fractionation factor that is simply described as:


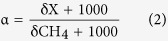


where δX = δ^13^C-DIC or δ^2^H-H_2_O and δCH_4_ = the δ^13^C-CH_4_ or δ^2^H-CH_4_, respectively: such that α_DIC-CH4_ = the carbon isotope fractionation factor, and α_H2O-CH4_ = the hydrogen isotope fractionation factor.

### Rayleigh equations

Where Rayleigh equations are presented, we use the Rayleigh equation described as:


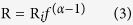


where R = change in isotope fractionation relative to the initial value, R_i_ = the initial isotope delta values, *f* = the residual reservoir (e.g. CH_4_). The use of the Rayleigh equation allows simple comparisons of the isotope partitioning as a defined reservoir (e.g. CH_4_) changes. Biogenic methanogenesis tends to operate at thermodynamic equilibrium, rather than being limited by kinetic controls^6^ so this approach is considered appropriate here, especially when working at large scales where multiple influences on the carbon and hydrogen isotope may occur. Here we use the Rayleigh equation to simply define the change in carbon and hydrogen isotope partition as CH_4_ changes relative to a CH_4_ end member (we do not propose that the δ^13^C-CH_4_ or δ^2^H-CH_4_ behaviour in the shallow coal measures always follows a simple Rayleigh fractionation process).

### Microbial pathways and Gibbs free energy values

Gibbs free energy values (ΔG°) were calculated for a number of microbial pathways (equations (6–8)) using enthalpy and entropy values for each reaction listed in Stumm and Morgan^88^ and corrected for the temperature of each sample (equation 4).


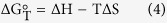


Where ΔH is the change in enthalpy and ΔS is the change in entropy for each reaction, and T is the temperature in Kelvin for each sample.

Changes in Gibbs Free Energy ΔG were calculated via equation (5).


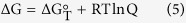


where R is the universal gas constant, T is the temperature in Kelvin and Q is the reaction quotient for each reaction.

For each reaction the activities of reactants and products [activity] were used to calculate Q. The activities of the reactants and products were calculated using *PHREEQC Interactive 3.1.7–9213* using the phreeqc.dat database.

The reaction pathways for CO_2_-reduction (hydrogenotrophic methanogenesis), SO_4_ reduction and anaerobic oxidation of CH_4_ (AOM) are as follows:


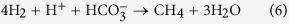


#### CO_2_ reduction



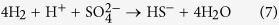



#### SO_4_ reduction



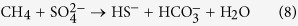



#### Anaerobic oxidation of CH_4_ (AOM)

Gibbs free energy values (ΔG^o^_T_) for the CO_2_-reduction pathways and SO_4_ reduction pathways were ~−229 kJ mol^−1^ and −262 kJ mol^−1^, respectively, which is consistent with calculations made for other studies^59^,^89^,^90^.

## Additional Information

**How to cite this article**: Owen, D. D. R. *et al.* Thermodynamic and hydrochemical controls on CH_4_ in a coal seam gas and overlying alluvial aquifer: new insights into CH_4_ origins. *Sci. Rep.* **6**, 32407; doi: 10.1038/srep32407 (2016).

## Supplementary Material

Supplementary Information

## Figures and Tables

**Table 1:** The applications of the combined approaches used to understand the origins and controls on CH_4_ in this study.

Approach	Application
δ^13^C-CH_4_ and δ^2^H-CH_4_	Collectively these two isotopes allow more informative assessments of potential CH_4_ origins than δ^13^C-CH_4_ alone. E.g. Acetoclastic methanogenesis produces an enriched δ^13^C-CH_4_ value that is similar to both CSG CH_4_ and other thermogenic CH_4_, but it can be distinguished from these by the highly depleted δ^2^H-CH_4_^3^,^26^.
Isotope fractionation factors α_DIC-CH4_ and α_H2O-CH4_	The ratio of isotope values between source carbon/hydrogen and that of CH_4_ provides insight into the production and consumption pathways^3^,^5^,^26^.
Thermodynamic data	Reducing organisms, such as SO_4_ reducers, operate at thermodynamic thresholds that inhibit less-competitive methanogenic processes^4^,^5^,^6^. Comparisons of Gibbs free energy values for production and consumption pathways provide information on the extent to which certain reaction processes have proceeded in the subsurface.
Isometric log ratios (compositional data analysis)	Isometric log ratios allow robust, simultaneous analysis of the ratios of parts and subparts, even where the concentration of subparts are very small^86^. When applied to the reaction pathways for key production and consumption reactions, isometric log ratios satisfy the law of mass balance and allow any rate-limiting effects associated with the availability of reactants to be elucidated.

**Table 2:** Sequential binary partition for the CO_2_ reduction pathway.

	[H^+^]	[H_2_]	[HCO_3_]	[CH_4_]
CO_2__ilr.1	−1	−1	−1	1
CO_2__ilr.2	−1	1	−1	
CO_2__ilr.3	−1		1	

**Table 3:** Sequential binary partition for the SO_4_ reduction pathway.

	[SO_4_]	[H_2_]	[H^+^]	[HS^−^]
SO_4__ilr.1	−1	−1	−1	1
SO_4__ilr.2	−1	1	−1	
SO_4__ilr.3	1		−1	

**Table 4:** Sequential binary partition for the anaerobic oxidation of CH_4_ (AOM) pathway.

	[HCO_3_]	[CH_4_]	[SO_4_]	[HS^−^]
AOM_ilr.1	1	−1	−1	1
AOM_ilr.2	1			−1
AOM_ilr.3		1	−1	

**Table 5:** Sequential binary partition of a four-part composition (x_1_, x_2_…….x_4_) deriving three orthonormal coordinates (z_1_, z_2_ and z_3_) for ilr calculation.

Balance	Partition of parts			
	x_1_	x_2_	x_3_	x_4_
z_1_	1	1	−1	−1
z_2_	1	−1		
Z_3_			−1	1

## References

[b1] Le Mer J. & Roger P. Production, oxidation, emission and consumption of methane by soils: A review. Eur J Soil Biol 37, 25–50, doi: http://dx.doi.org/10.1016/S1164-5563(01)01067-6 (2001).

[b2] Kotelnikova S. Microbial production and oxidation of methane in deep subsurface. Earth-Sci. Rev. 58, 367–395, doi: http://dx.doi.org/10.1016/S0012-8252(01)00082-4 (2002).

[b3] Whiticar M. J. Carbon and hydrogen isotope systematics of bacterial formation and oxidation of methane. Chem. Geol. 161, 291–314, doi: http://dx.doi.org/10.1016/S0009-2541(99)00092-3 (1999).

[b4] Cord-Ruwisch R., Seitz H.-J. & Conrad R. The capacity of hydrogenotrophic anaerobic bacteria to compete for traces of hydrogen depends on the redox potential of the terminal electron acceptor. Arch. Microbiol. 149, 350–357, doi: 10.1007/BF00411655 (1988).

[b5] Conrad R. Quantification of methanogenic pathways using stable carbon isotopic signatures: a review and a proposal. Org. Geochem. 36, 739–752, doi: http://dx.doi.org/10.1016/j.orggeochem.2004.09.006 (2005).

[b6] Conrad R. Contribution of hydrogen to methane production and control of hydrogen concentrations in methanogenic soils and sediments. FEMS Microbiol. Ecol. 28, 193–202, doi: 10.1111/j.1574-6941.1999.tb00575.x (1999).

[b7] Chanton J. P., Fields D. & Hines M. E. Controls on the hydrogen isotopic composition of biogenic methane from high-latitude terrestrial wetlands. J. Geophys. Res. (G Biogeosci) 111, n/a-n/a, doi: 10.1029/2005JG000134 (2006).

[b8] Kinnaman F. S., Valentine D. L. & Tyler S. C. Carbon and hydrogen isotope fractionation associated with the aerobic microbial oxidation of methane, ethane, propane and butane. Geochim. Cosmochim. Acta 71, 271–283, doi: http://dx.doi.org/10.1016/j.gca.2006.09.007 (2007).

[b9] Botz R., Pokojski H.-D., Schmitt M. & Thomm M. Carbon isotope fractionation during bacterial methanogenesis by CO_2_ reduction. Org. Geochem. 25, 255–262, doi: http://dx.doi.org/10.1016/S0146-6380(96)00129-5 (1996).

[b10] Strąpoć D., Schimmelmann A. & Mastalerz M. Carbon isotopic fractionation of CH_4_ and CO_2_ during canister desorption of coal. Org. Geochem. 37, 152–164, doi: http://dx.doi.org/10.1016/j.orggeochem.2005.10.002 (2006).

[b11] Xia X. & Tang Y. Isotope fractionation of methane during natural gas flow with coupled diffusion and adsorption/desorption. Geochim. Cosmochim. Acta 77, 489–503, doi: http://dx.doi.org/10.1016/j.gca.2011.10.014 (2012).

[b12] Prinzhofer A. & Pernaton É. Isotopically light methane in natural gas: bacterial imprint or diffusive fractionation? Chem. Geol. 142, 193–200, doi: http://dx.doi.org/10.1016/S0009-2541(97)00082-X (1997).

[b13] Whiticar M. J., Faber E. & Schoell M. Biogenic methane formation in marine and freshwater environments: CO_2_ reduction vs. acetate fermentation–Isotope evidence. Geochim. Cosmochim. Acta 50, 693–709, doi: http://dx.doi.org/10.1016/0016-7037(86)90346-7 (1986).

[b14] Heimann A., Jakobsen R. & Blodau C. Energetic Constraints on H2-Dependent Terminal Electron Accepting Processes in Anoxic Environments: A Review of Observations and Model Approaches. Environ. Sci. Technol. 44, 24–33, doi: 10.1021/es9018207 (2010). 20039730

[b15] Penger J., Conrad R. & Blaser M. Stable carbon isotope fractination by methyltrophic methanogenic archea. Applied Environmental Microbiology 78, 7596–7602, doi: doi: 10.1128/AEM.01773-12 (2012). 22904062 PMC3485729

[b16] Roy R., Klüber H. D. & Conrad R. Early initiation of methane production in anoxic rice soil despite the presence of oxidants. FEMS Microbiol. Ecol. 24, 311–320, doi: http://dx.doi.org/10.1016/S0168-6496(97)00072-X (1997).

[b17] Etiope G. Natural Gas Seepage. Vol. 1, Ch. 3, 50–52 (Springer International Publishing 2015).

[b18] McIntosh J. S., M. & Bates B. In Technical Workshops for the hydraulic fracturing study: US EPA, Feb 24–25, 2011. (United States Environmental Protection Agency).

[b19] Aravena R., Harrison S. M., Barker J. F., Abercrombie H. & Rudolph D. Origin of methane in the Elk Valley coalfield, southeastern British Columbia, Canada. Chem. Geol. 195, 219–227, doi: 10.1016/s0009-2541(02)00396-0 (2003).

[b20] Hansen L. K., Jakobsen R. & Postma D. Methanogenesis in a shallow sandy aquifer, Rømø, Denmark. Geochim. Cosmochim. Acta 65, 2925–2935, doi: http://dx.doi.org/10.1016/S0016-7037(01)00653-6 (2001).

[b21] Iverach C. P. *et al.* Assessing Connectivity Between an Overlying Aquifer and a Coal Seam Gas Resource Using Methane Isotopes, Dissolved Organic Carbon and Tritium. Sci Rep. 5, 15996, doi: 10.1038/srep15996, http://www.nature.com/articles/srep15996#supplementary-information (2015). 26530701 PMC4632156

[b22] Huxley W. J. *The hydrogeology, hydrology and hydrochemistry of the Condamine River Valley Alluvium* Masters thesis, Queensland Institute of Technology (1982).

[b23] Owen D. D. R. & Cox M. E. Hydrochemical evolution within a large alluvial groundwater resource overlying a shallow coal seam gas reservoir. Sci. Total Environ. 523, 233–252, doi: http://dx.doi.org/10.1016/j.scitotenv.2015.03.115 (2015). 25863513 10.1016/j.scitotenv.2015.03.115

[b24] Dafny E. & Silburn D. M. The hydrogeology of the Condamine River Alluvial Aquifer, Australia: a critical assessment. Hydrogeol. J., 1–23, doi: 10.1007/s10040-013-1075-z (2013).

[b25] QWC. (ed Queensland Water Commission) (Brisbane, 2012).

[b26] Golding S. D., Boreham C. J. & Esterle J. S. Stable isotope geochemistry of coal bed and shale gas and related production waters: A review. Int. J. Coal Geol. 120, 24–40, doi: http://dx.doi.org/10.1016/j.coal.2013.09.001 (2013).

[b27] Baublys K. A., Hamilton S. K., Golding S. D., Vink S. & Esterle J. Microbial controls on the origin and evolution of coal seam gases and production waters of the Walloon Subgroup; Surat Basin, Australia. Int. J. Coal Geol. 147–148, 85–104, doi: http://dx.doi.org/10.1016/j.coal.2015.06.007 (2015).

[b28] Hamilton S. K., Golding S. D., Baublys K. A. & Esterle J. S. Stable isotopic and molecular composition of desorbed coal seam gases from the Walloon Subgroup, eastern Surat Basin, Australia. Int. J. Coal Geol. 122, 21–36, doi: http://dx.doi.org/10.1016/j.coal.2013.12.003 (2014).

[b29] Draper J. J. & Boreham C. J. Geological controls on exploitable coal seam gas distribution in Queensland. APPEA Journal 46, 343–366. (2006).

[b30] Cook A. G. & Draper J. J. In Geology of Queensland (ed. Jell P. A.) Ch. 7, 533–539 (Geological Survey of Queensland, Brisbane, QLD, 2013).

[b31] Jell P. A., McKellar J. L. & Draper J. J. Geology of Queensland: 7.10 Clarence-Moreton Basin. 5 (2013).

[b32] Owen D. D. R., Millot R., Négrel P., Meredith K. & Cox M. E. Stable Isotopes of Lithium as Indicators of Coal Seam Gas-bearing Aquifers. Procedia Earth Planet. Sci. 13, 278–281, doi: http://dx.doi.org/10.1016/j.proeps.2015.07.065 (2015).

[b33] Richards L. A., Magnone D., van Dongen B. E., Ballentine C. J. & Polya D. A. Use of lithium tracers to quantify drilling fluid contamination for groundwater monitoring in Southeast Asia. Appl. Geochem. 63, 190–202, doi: http://dx.doi.org/10.1016/j.apgeochem.2015.08.013 (2015).

[b34] Murray J. P., Rouse J. V. & Carpenter A. B. Groundwater contamination by sanitary landfill leachate and domestic wastewater in carbonate terrain: Principal source diagnosis, chemical transport characteristics and design implications. Water Res. 15, 745–757, doi: http://dx.doi.org/10.1016/0043-1354(81)90168-8 (1981).

[b35] Carrillo-Rivera J. J., Cardona A. & Edmunds W. M. Use of abstraction regime and knowledge of hydrogeological conditions to control high-fluoride concentration in abstracted groundwater: San Luis Potosí basin, Mexico. J Hydrol 261, 24–47, doi: 10.1016/S0022-1694(01)00566-2 (2002).

[b36] Hem J. D. Study and interpretation of the chemical characteristics of natural water: USGS Water-Supply Paper 2254. Third edn (United States Geological Survey, 1985).

[b37] Wrenn B. A. *et al.* Nutrient transport during bioremediation of contaminated beaches: Evaluation with lithium as a conservative tracer. Water Res. 31, 515–524, doi: http://dx.doi.org/10.1016/S0043-1354(96)00304-1 (1997).

[b38] Humez P., Mayer B., Nightingale M., Becker V., Kingston A., Taylor S., Bayegnak G., Millot R. & Kloppmann W. Redox controls on methane formation, migration and fate in shallow aquifers. Hydrol. Earth Syst. Sci. Discuss., doi: 10.5194/hess-2016-85, in review, 2016 (2016).

[b39] Aravena R. & Wassenaar L. I. Dissolved organic carbon and methane in a regional confined aquifer, southern Ontario, Canada: Carbon isotope evidence for associated subsurface sources. Appl. Geochem. 8, 483–493, doi: http://dx.doi.org/10.1016/0883-2927(93)90077-T (1993).

[b40] Wassenaar L., Aravena R., Hendry J. & Fritz P. Radiocarbon in Dissolved Organic Carbon, A Possible Groundwater Dating Method: Case Studies From Western Canada. Water Resour. Res. 27, 1975–1986, doi: 10.1029/91WR00504 (1991).

[b41] Flores R. M., Rice C. A., Stricker G. D., Warden A. & Ellis M. S. Methanogenic pathways of coal-bed gas in the Powder River Basin, United States: The geologic factor. Int. J. Coal Geol. 76, 52–75, doi: http://dx.doi.org/10.1016/j.coal.2008.02.005 (2008).

[b42] Blair N. E. & Carter W. D. Jr. The carbon isotope biogeochemistry of acetate from a methanogenic marine sediment. Geochim. Cosmochim. Acta 56, 1247–1258, doi: http://dx.doi.org/10.1016/0016-7037(92)90060-V (1992).

[b43] Riveros-Iregui D. A. & King J. Y. Isotopic evidence of methane oxidation across the surface water-ground water interface. Wetlands 28, 928–937, doi: 10.1672/07-191.1 (2008).

[b44] Moura J. M. S. *et al.* Spatial and seasonal variations in the stable carbon isotopic composition of methane in stream sediments of eastern Amazonia. Tellus B 60, 21–31, doi: 10.1111/j.1600-0889.2007.00322.x (2008).

[b45] Tsunogai U., Yoshida N. & Gamo T. Carbon isotopic evidence of methane oxidation through sulfate reduction in sediment beneath cold seep vents on the seafloor at Nankai Trough. Mar. Geol. 187, 145–160, doi: http://dx.doi.org/10.1016/S0025-3227(02)00263-3 (2002).

[b46] Ahmed M. & Smith J. W. Biogenic methane generation in the degradation of eastern Australian Permian coals. Org. Geochem. 32, 809–816, doi: http://dx.doi.org/10.1016/S0146-6380(01)00033-X (2001).

[b47] Chanton J. P., Chasar L. C., Glaser P. & Siegel D. In Stable Isotopes and Biosphere-Atmosphere Interactions, Physiol. Ecol. Ser. (eds Flanagan L. B., Ehleringer J. R. & Pataki D. E.) Ch. 6, 85–105 (Elsevier, 2005).

[b48] Waldron S., Lansdown J. M., Scott E. M., Fallick A. E. & Hall A. J. The global influence of the hydrogen iostope composition of water on that of bacteriogenic methane from shallow freshwater environments. Geochim. Cosmochim. Acta 63, 2237–2245, doi: http://dx.doi.org/10.1016/S0016-7037(99)00192-1 (1999).

[b49] Papendick S. L. *et al.* Biogenic methane potential for Surat Basin, Queensland coal seams. Int. J. Coal Geol. 88, 123–134, doi: http://dx.doi.org/10.1016/j.coal.2011.09.005 (2011).

[b50] McIntosh J. C., Grasby S. E., Hamilton S. M. & Osborn S. G. Origin, distribution and hydrogeochemical controls on methane occurrences in shallow aquifers, southwestern Ontario, Canada. Appl. Geochem. 50, 37–52, doi: http://dx.doi.org/10.1016/j.apgeochem.2014.08.001 (2014).

[b51] Quillinan S. A. & Frost C. D. Carbon isotope characterization of powder river basin coal bed waters: Key to minimizing unnecessary water production and implications for exploration and production of biogenic gas. Int. J. Coal Geol. 126, 106–119, doi: http://dx.doi.org/10.1016/j.coal.2013.10.006 (2014).

[b52] Clark I. & Fritz P. Environmental Isotopes in Hydrogeology. (CRC Press LLC, 1997).

[b53] Lovley D. R. & Klug M. J. Sulfate Reducers Can Outcompete Methanogens at Freshwater Sulfate Concentrations. Appl. Environ. Microbiol. 45, 187–192 (1983). 16346164 10.1128/aem.45.1.187-192.1983 PMC242251

[b54] Stams A. J. M. *et al.* Metabolic interactions in methanogenic and sulfate-reducing bioreactors. Water Sci. Technol. 52, 13–20 (2005). 16187442

[b55] Kato M. T., Field J. A. & Lettinga G. The anaerobic treatment of low strength wastewaters in UASB and EGSB reactors. Water Sci. Technol. 36, 375–382, doi: http://dx.doi.org/10.1016/S0273-1223(97)00545-3 (1997).

[b56] Walker G. R. & Mallants D. Methodologies for Investigating Gas in Water Bores and Links to Coal Seam Gas Development. (Australia, 2014).

[b57] Feitz A. J. *et al.* Geoscience Australia and Geological Survey of Queensland Surat and Bowen Basins Groundwater Surveys Hydrochemistry Dataset (2009–2011). (Canberra Australia, 2014).

[b58] Lovley D. R. & Goodwin S. Hydrogen concentrations as an indicator of the predominant terminal electron-accepting reactions in aquatic sediments. Geochim. Cosmochim. Acta 52, 2993–3003, doi: http://dx.doi.org/10.1016/0016-7037(88)90163-9 (1988).

[b59] Strąpoć D. *et al.* Methane-producing microbial community in a coal bed of the Illinois basin. Appl. Environ. Microbiol. 74, 2424–2432, doi: 10.1128/AEM.02341-07 (2008). 18310416 PMC2293134

[b60] Egozcue J. J. & Pawlowsky-Glahn V. Groups of Parts and Their Balances in Compositional Data Analysis. Math. Geol. 37, 795–828, doi: 10.1007/s11004-005-7381-9 (2005).

[b61] Smemo K. A. & Yavitt J. B. Evidence for Anaerobic CH_4_ Oxidation in Freshwater Peatlands. Geomicrobiol. J. 24, 583–597, doi: 10.1080/01490450701672083 (2007).

[b62] Games L. M., HayesRobert J. M. & Gunsalus P. Methane-producing bacteria: natural fractionations of the stable carbon isotopes. Geochim. Cosmochim. Acta 42, 1295–1297, doi: http://dx.doi.org/10.1016/0016-7037(78)90123-0 (1978).

[b63] Krzycki J. A., Kenealy W. R., DeNiro M. J. & Zeikus J. G. Stable Carbon Isotope Fractionation by Methanosarcina barkeri during Methanogenesis from Acetate, Methanol, or Carbon Dioxide-Hydrogen. Appl. Environ. Microbiol. 53, 2597–2599 (1987). 16347476 10.1128/aem.53.10.2597-2599.1987 PMC204153

[b64] Balabane M., Galimov E., Hermann M. & Létolle R. Hydrogen and carbon isotope fractionation during experimental production of bacterial methane. Org. Geochem. 11, 115–119, doi: http://dx.doi.org/10.1016/0146-6380(87)90033-7 (1987).

[b65] Alperin M. J., Blair N. E., Albert D. B., Hoehler T. M. & Martens C. S. Factors that control the stable carbon isotopic composition of methane produced in an anoxic marine sediment. Global Biogeochem. Cycles 6, 271–291, doi: 10.1029/92GB01650 (1992).

[b66] Bilek R. S., Tyler S. C., Sass R. L. & Fisher F. M. Differences in CH4 oxidation and pathways of production between rice cultivars deduced from measurements of CH_4_ flux and δ13C of CH4 and CO2. Global Biogeochem. Cycles 13, 1029–1044, doi: 10.1029/1999GB900040 (1999).

[b67] Sass R. L., Fisher F. M., Lewis S. T., Jund M. F. & Turner F. T. Methane emissions from rice fields: Effect of soil properties. Global Biogeochem. Cycles 8, 135–140, doi: 10.1029/94GB00588 (1994).

[b68] Oades J. M. The Retention of Organic Matter in Soils. Biogeochemistry 5, 35–70 (1988).

[b69] Kits K. D., Klotz M. G. & Stein L. Y. Methane oxidation coupled to nitrate reduction under hypoxia by the Gammaproteobacterium Methylomonas denitrificans, sp. nov. type strain FJG1. Environ. Microbiol. 17, 3219–3232, doi: 10.1111/1462-2920.12772 (2015). 25580993

[b70] Ettwig K. F. *et al.* Denitrifying bacteria anaerobically oxidize methane in the absence of Archaea. Environ. Microbiol. 10, 3164–3173, doi: 10.1111/j.1462-2920.2008.01724.x (2008). 18721142

[b71] Conrad R., Klose M., Claus P. & Enrich-Prast A. Methanogenic pathway, 13C isotope fractionation, and archaeal community composition in the sediment of two clear-water lakes of Amazonia. Limnol. Oceanogr. 55, 689–702, doi: 10.4319/lo.2010.55.2.0689 (2010).

[b72] Schlegel M. E., McIntosh J. C., Bates B. L., Kirk M. F. & Martini A. M. Comparison of fluid geochemistry and microbiology of multiple organic-rich reservoirs in the Illinois Basin, USA: Evidence for controls on methanogenesis and microbial transport. Geochim. Cosmochim. Acta 75, 1903–1919, doi: http://dx.doi.org/10.1016/j.gca.2011.01.016 (2011).

[b73] Morgenstern U. & Taylor C. B. Ultra low-level tritium measurement using electrolytic enrichment and LSC. Isotopes Environ. Health Stud. 45, 96–117, doi: 10.1080/10256010902931194 (2009). 20183224

[b74] Hocking M. & Kelly B. F. J. Groundwater recharge and time lag measurement through Vertosols using impulse response functions. J Hydrol 535, 22–35, doi: http://dx.doi.org/10.1016/j.jhydrol.2016.01.042 (2016).

[b75] KCB. Central Condamine alluvium, stage III: detailed water balance: Final Report. (Toowoomba, Queensland, 2011).

[b76] Hendry M. J., Ranville J. R., Boldt-Leppin B. E. J. & Wassenaar L. I. Geochemical and transport properties of dissolved organic carbon in a clay-rich aquitard. Water Resour. Res. 39, n/a-n/a, doi: 10.1029/2002WR001943 (2003).

[b77] Sharma M. L. & Hughes M. W. Groundwater recharge estimation using chloride, deuterium and oxygen-18 profiles in the deep coastal sands of Western Australia. J Hydrol 81, 93–109, doi: http://dx.doi.org/10.1016/0022-1694(85)90169-6 (1985).

[b78] Dlugokencky E. J., Nisbet E. G., Fisher R. & Lowry D. Global atmospheric methane: budget, changes and dangers. Philos. Trans. Roy. Soc. London Ser. A 369, 2058–2072, doi: 10.1098/rsta.2010.0341 (2011). 21502176

[b79] Stalker L. Methane origins and behaviour (Commonwealth Scientific and Industrial Research Organisation, Australia, 2013).

[b80] Khalil M. A. K. Atmospheric Methane: Sources, Sinks, and Role in Global Change. 199–229 (Springer-Verlag, 1991).

[b81] Puls R. W. & Barcelona M. J. LOW-FLOW (MINIMAL DRAWDOWN) GROUND-WATER SAMPLING PROCEDURES (United States Environmental Protection Agency, 1996).

[b82] Barcelona M. J., Varljen M. D., Puls R. W. & Kaminski D. Ground water purging and sampling methods: History vs. hysteria. Ground Water Monitoring & Remediation 25, 52–62, doi: 10.1111/j.1745-6592.2005.0001.x (2005).

[b83] Noble R. R. P., Gray D. J. & Gill A. J. Field guide for mineral exploration using hydrogeochemical analysis (Bentley, Western Australia, 2011).

[b84] Peacock M., Freeman C., Gauci V., Lebron I. & Evans C. D. Investigations of freezing and cold storage for the analysis of peatland dissolved organic carbon (DOC) and absorbance properties. Environmental Science: Processes & Impacts 17, 1290–1301, doi: 10.1039/C5EM00126A (2015). 26051006

[b85] Palarea-Albaladejo J. & Martín-Fernández J. A. zCompositions—R package for multivariate imputation of left-censored data under a compositional approach. Chemometrics Intellig. Lab. Syst. 143, 85–96, doi: http://dx.doi.org/10.1016/j.chemolab.2015.02.019 (2015).

[b86] Egozcue J. J., Pawlowsky-Glahn V., Mateu-Figueras G. & Barceló-Vidal C. Isometric Logratio Transformations for Compositional Data Analysis. Math. Geol. 35, 279–300, 10.1023/A:1023818214614 (2003).

[b87] Comas-Cufí M. & Thió-Henestrosa S. In CoDaWork'11: 4th International Workshop on Compositional Data (eds Egozcue J. J., Tolosana-Delgado R. & Ortego M. I.) (2011).

[b88] Stumm W. & Morgan J. J. Aquatic chemistry: chemical equilibria and rates in natural waters. Third Edition edn, 1022 (John wiley and Sons, Inc, 1996).

[b89] Lin H.-T. *et al.* Dissolved hydrogen and methane in the oceanic basaltic biosphere. Earth. Planet. Sci. Lett. 405, 62–73, doi: http://dx.doi.org/10.1016/j.epsl.2014.07.037 (2014).

[b90] Ozuolmez D. *et al.* Methanogenic archaea and sulfate reducing bacteria co-cultured on acetate: teamwork or coexistence? Frontiers in Microbiology 6, 492, doi: 10.3389/fmicb.2015.00492 (2015). 26074892 PMC4445324

[b91] Cox M. E., James A., Hawke A. & Raiber M. Groundwater Visualisation System (GVS): A software framework for integrated display and interrogation of conceptual hydrogeological models, data and time-series animation. J Hydrol 491, 56–72, doi: http://dx.doi.org/10.1016/j.jhydrol.2013.03.023 (2013).

[b92] Lane W. B. Progress Report on Condamine Underground Investigation to December 1978 (Brisbane, Queensland, 1979).

[b93] Crosbie R. S. *et al.* (ed. CSIRO Water for a Healthy Country Flagship) (Australia 2012).

