# Supplementary material for: Thermodynamic and hydrochemical controls on CH4 in a coal seam gas and overlying alluvial aquifer: new insights into CH4 origins
Source: Sci Rep. 2016 Aug 31;6:32407. doi: 10.1038/srep32407 (PMC5006171; doi:10.1038/srep32407)
Supplement: Supplementary Information [file srep32407-s1.pdf]

Thermodynamic and hydrochemical controls on CH_4 in a coal seam gas and overlying alluvial aquifer: new insights into CH_4 origins

D. Des. R. Owen[1], Shouakar-Stash, O.[2], Morgenstern, U.[3], Aravena, R[4].*

[1]*School of Earth, Environmental and Biological Sciences, Queensland University of Technology, Brisbane, Queensland, 4000, Australia*
[2]*Isotope Tracer Technologies, Waterloo, ON N2V 1Z5, Canada*
[3]*GNS Science, Lower Hutt 5014, P.O. Box 30368, New Zealand*
[4]*Department of Earth and Environmental Sciences, University of Waterloo, Ontario N2L 3G1, Canada*

**corresponding author Des Owen* dr.owen@qut.edu.au

Supporting Information

Introduction. This supporting information includes Figure S1, which outlines the ranges of $\delta^{13}C$-CH_4 values for different CH_4 sources, environments and processes, Figure S2 which shows a map of wells sampled in this study; and Tables S1 – S4, which provide the raw data used in the study.

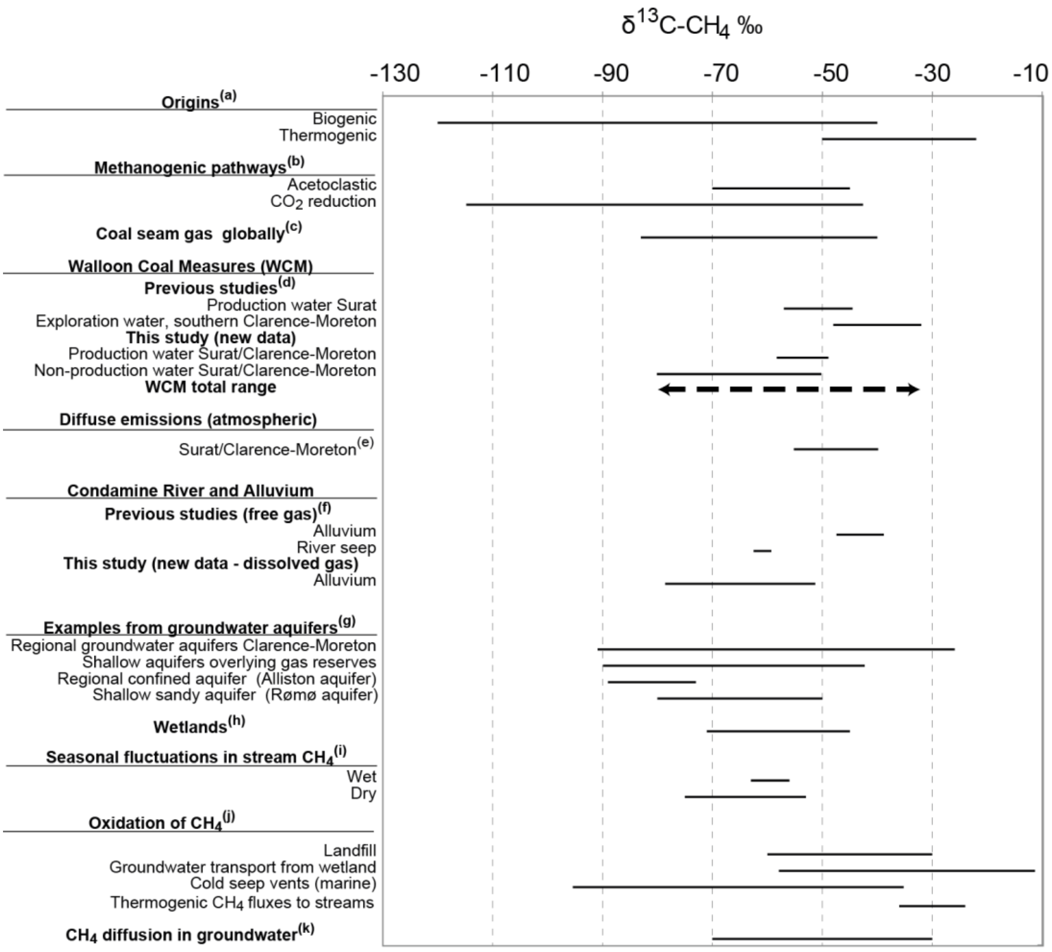

Figure S1. Ranges of $\delta^{13}C$-CH_4 under various pathways, conditions and processes as well as in different basins/sedimentary formations and aquifers. The ranges for oxidation and diffusion report the ranges of $\delta^{13}C$-CH_4 observed in the relevant studies for each process. References are as follows: (a) [1-5]; (b) [1,2,5-7]; (c) [8-12]; (d) [13-17] ; (e) [18]; (f) [19,20]; (g) [12,21-24]; (h) [25]; (i) [26]; (j) [27-32]; (k) [33]. The result highlights the large and overlapping range of $\delta^{13}C$-CH_4. As a result $\delta^{13}C$-CH_4 should not be used as a "signature" or "tracer" of CH_4 source or in isolation when assessing potential CH_4 migration.

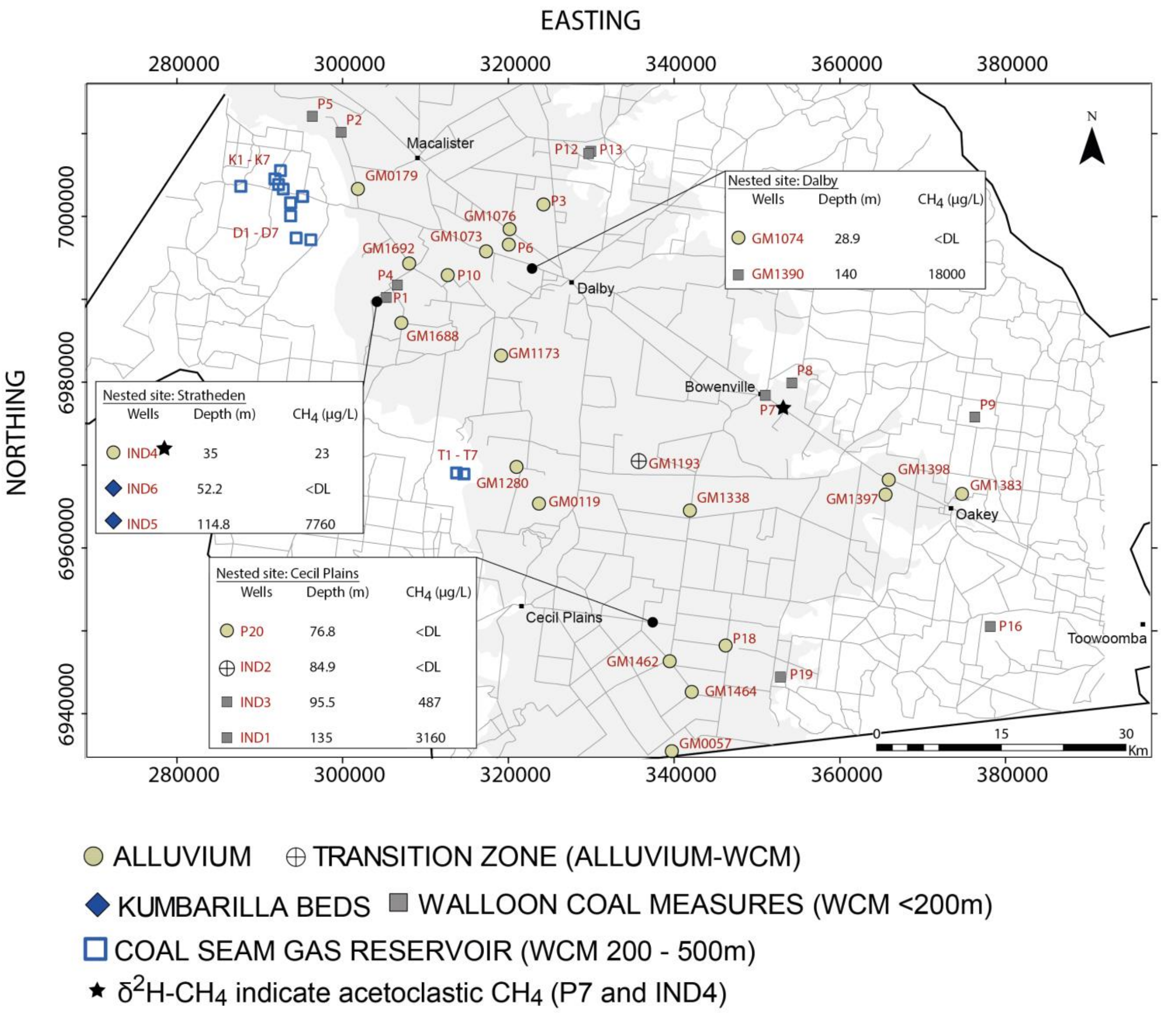

Figure S2. Sample locations and well IDs for all wells sampled in this study. The map was prepared using *ArcGIS v 10.1* (www.esri.com) and modified using *Adobe Illustrator CC 2014.* Grey lines represent the road network as provided by GeoScience Australia (National Geoscience datasets: 34877)[34] and after modification by QUT for the QUT Groundwater Visualisation System. CH_4 DL = 10 µg/L.

Table S1. Hydrochemical (dissolved) and CH_4 data for all wells with dissolved CH_4 > DL (10 µg/L).

Sample	Depth	Aquifer	nested site	pH	Temp	DO	TDS	HCO_3	CO_3	SO_4	Cl	Ca	Mg	Na	K	Br	NO_3	Fe total	Mn total	DOC	DOC_{adj}	CH_4	Ethene	Ethane	Propene	SI kaolinite	SI gypsum
	m				°C	mg/L	mg/L	meq/L	meq/L	meq/L	meq/L	meq/L	meq/L	meq/L	meq/L	meq/L	meq/L	mg/L	mg/L	mg/L	mg/L	µg/L	µg/L	µg/L	µg/L		
GM1076	27.7	Alluvium		7.3	20.5	2.18	1480	7.70		1.40	12.51	1.50	2.30	18.83	0.10	0.02	3.14E-02	<0.05	0.001	0.17	0.23	10	< 10	< 10	< 10	3.63	-2.31
IND4	35	Alluvium	Stratheden	6.8	22.4	1.55	10200	5.52		2.92	168.73	52.89	33.90	72.61	0.23	0.20	< 1.62E-04	4.86	3.961	3.90	4.53	23	< 10	< 10	< 10	6.06	-1.15
GM1073	18.3	Alluvium		7.09	21.8	0.41	503	4.74		0.23	2.37	0.50	0.58	6.48	0.05	0.00	< 1.62E-04	0.11	0.183	0.40	0.56	171	< 10	< 10	< 10	4.94	-3.33
GM0057	56.7	Alluvium		7.04	20.3	4.51	680	5.44		0.04	4.68	3.59	2.63	3.78	0.05	0.01	4.19E-03	<0.05	<0.001	0.28	0.38	535	< 10	< 10	< 10	5.86	-3.34
GM1193	110	Alluv-WCM		7.17	21.1	3.16	1590	5.96		0.92	16.25	1.55	3.29	18.30	0.10	0.03	3.55E-03	<0.05	<0.001	0.40	0.55	218	< 10	< 10	< 10	3.57	-2.48
P7	60.4	WCM		6.84	22.1	3.60	4560	8.16		10.17	48.73	12.43	11.85	46.52	0.20	0.07	< 1.62E-04	19.42	0.324	0.31	0.42	95	< 10	< 10	< 10	4.32	-0.91
IND3	95.5	WCM	Cecil Plains	7.22	32.4	0.19	786	4.18		0.25	7.30	2.54	1.97	6.74	0.26	0.01	< 1.62E-04	0.56	0.021	1.63	2.19	487	25	15	< 10	2.78	-2.72
P19	185.9	WCM		8.11	28	2.46	2200	6.18	1.41	1.00	23.46	0.90	0.66	31.61	0.10	0.04	3.23E-04	0.06	0.014	0.26	0.35	1390	< 10	< 10	< 10	0.92	-2.73
IND1	135	WCM	Cecil Plains	8.55	27.9	0.00	897	8.14	2.61	0.17	4.56	0.60	0.25	13.30	0.18	0.01	< 1.62E-04	0.79	0.068			3160	70	30	24	-0.47	-3.54
P4	82.9	WCM		7.46	22.8	1.52	2020	4.74	0.00	<0.02	22.90	3.04	3.46	22.26	0.10	0.04	< 1.62E-04	0.38	0.090	0.90	1.25	6850	< 10	< 10	< 10	3.12	-4.03
P1	100.9	WCM		7.84	21.1	0.04	1430	5.40	0.44	<0.02	15.24	1.20	1.15	18.91	0.05	0.02	< 1.62E-04	0.44	0.024	0.48	0.68	12300	< 10	< 10	< 10	2.16	-4.23
GM1390	140	WCM	Dalby	7.98	20.3	0.51	2180	17.72	1.54	<0.02	15.15	0.25	0.33	31.78	0.08	0.00	< 1.62E-04	<0.05	0.001	0.33	0.46	18000	< 10	< 10	< 10	2.07	-5.03
IND5	114.8	Kumbarilla	Stratheden	11.55*	20.9	1.04	2280		1.61	0.35	28.73	2.40	0.04	26.04	0.20	0.05	< 1.62E-04	<0.05	0.004			7760	< 10	< 10	< 10		
D1	-	CSG		7.75	27.9	4.58	8900	5.48		<0.02	126.20	2.40	1.23	123.91	0.31		< 1.62E-04	0.06	0.053			1860	< 10	< 10	< 10		
D2	-	CSG		8.26	29.2	4.11	4850	17.58	1.84	<0.02	56.34	0.30	0.25	70.43	0.15		< 1.62E-04	<0.05	0.007			3870	< 10	< 10	< 10		
D3	-	CSG		8.45	30.1	4.59	3870	21.60	3.69	<0.02	30.70	0.25	0.16	59.57	0.13		< 1.62E-04	<0.05	0.005			2040	< 10	< 10	< 10		
D4	-	CSG		8.58	28.1	1.07	3670	20.80	4.25	<0.02	28.73	0.20	0.16	56.09	0.13		< 1.62E-04	<0.05	0.004			21500	< 10	< 10	< 10		
D5	-	CSG		7.98	28.3	0.47	6060	13.44	0.54	<0.02	76.90	0.65	0.49	89.13	0.20		< 1.62E-04	0.07	0.024			22000	< 10	< 10	< 10		
D6	-	CSG		8.3	31	0.54	4620	24.80	2.95	<0.02	47.32	0.20	0.16	71.30	0.15		< 1.62E-04	<0.05	0.006			18400	< 10	< 10	< 10		
D7	-	CSG		8.14	30.5	0.49	4420	22.80	1.64	<0.02	42.82	0.25	0.16	69.13	0.15		< 1.62E-04	<0.05	0.012			19400	< 10	< 10	< 10		
K1	-	CSG		7.81	29.5	1.19	5840	11.34	0.40	<0.02	82.82	0.70	0.49	85.65	0.18		< 1.62E-04	<0.05	0.007			14100	< 10	< 10	< 10		
K2	-	CSG		8.58	32.6	0.56	3450	25.40	6.57	<0.02	23.10	0.20	0.08	56.96	0.13		< 1.62E-04	<0.05	0.003			7840	< 10	< 10	< 10		
K3	-	CSG		8.06	29.3	4.49	5780	10.68	0.94	<0.02	74.93	0.65	0.41	86.96	0.20		< 1.62E-04	<0.05	0.007			5550	< 10	< 10	< 10		
K4	-	CSG		8	32.8	3.07	6760	8.60	0.60	<0.02	89.01	0.85	0.49	102.61	0.23		< 1.62E-04	0.48	0.012			5160	< 10	< 10	< 10		
K5	-	CSG		8.22	31.3	3.09	3920	21.80	2.95	<0.02	35.49	0.20	0.16	63.04	0.15		< 1.62E-04	<0.05	0.002			7990	< 10	< 10	< 10		
K6	-	CSG		7.89	29.8	1.83	6560	7.86	0.00	<0.02	88.45	0.80	0.49	93.91	0.20		< 1.62E-04	0.52	0.023			24800	< 10	< 10	< 10		
K7	-	CSG		8.35	30.7	1.25	4120	21.80	3.72	<0.02	39.15	0.20	0.16	63.91	0.15		< 1.62E-04	<0.05	0.006			21000	< 10	< 10	< 10		
T1	-	CSG		8.15	29.5	3.12	4020	16.60	1.24	<0.02	44.79	0.30	0.25	60.00	0.13		< 1.62E-04	<0.05	0.008			11100	< 10	< 10	< 10		
T2	-	CSG		8.49	32.2	0.94	3950	23.20	4.62	<0.02	30.14	0.20	0.16	60.00	0.13		< 1.62E-04	<0.05	0.003			17700	< 10	< 10	< 10		
T3	-	CSG		7.97	27.9	0.16	4080	15.26	0.90	<0.02	47.32	0.35	0.25	62.17	0.15		< 1.62E-04	<0.05	0.036			8640	< 10	< 10	< 10		
T4	-	CSG		8.34	34.2	0.49	3750	24.60	3.55	<0.02	31.27	0.20	0.08	61.30	0.13		< 1.62E-04	<0.05	0.005			18300	< 10	< 10	< 10		
T5	-	CSG		8.45	29	4.18	3930	16.20	2.95	<0.02	39.72	0.30	0.25	60.87	0.15		< 1.62E-04	<0.05	0.005			6140	< 10	< 10	< 10		
T6	-	CSG		8.43	34.1	3.98	4030	24.60	5.06	<0.02	30.14	0.20	0.16	60.87	0.13		3.23E-04	<0.05	0.002			2260	< 10	< 10	< 10		
T7	-	CSG		8	29.7	1.23	4970	16.06	1.21	<0.02	50.70	0.45	0.33	73.04	0.18		< 1.62E-04	<0.05	0.022			17600	< 10	< 10	< 10		

* possible measurement error (IND5), corrected pH in laboratory post sampling = ~ 8.55. WCM = Walloon Coal Measures. Alluv-WCM = sample at the alluvial basement/alluvial-coal measure interface. CSG = CSG production water sample taken from the relatively deep gas reservoir (200-500 m) in the WCM. DOC_{adj} calculated as per Peacock et al.[35]. NB: DO mg/L for CSG groundwater at some sites is not likely to be representative due to expulsion of water from extraction point causing splashing. SO_4 < DL (0.02 meq/L or 1 mg/L) at all CSG sites indicated highly reduced conditions. SI = saturation index

Table S2. Isotopic data for all wells with dissolved CH_4 >DL (10 µg/L).

Sample	Depth (m)	Aquifer	δ^2H‰	$\delta^{18}O$‰	δ^2H-CH_4‰	$\delta^{13}C-CH_4$‰	$\delta^{13}C$-DIC‰	$a_{DIC-CH4}$	$a_{H2O-CH4}$	$\delta^{37}Cl$‰	^3H (TU)
GM1076	27.7	Alluvium	-26.85	-4.15	-244.57	-51.38	-6.57	1.05	1.29	-0.72	0.05
IND4	35	Alluvium	-27.61	-4.29	-314.60	-49.81	-15.28	1.04	1.42	0.89	
GM1073	18.3	Alluvium	-38.16	-5.87	-186.38	-53.05	-13.12	1.04	1.18	0.22	
GM0057	56.7	Alluvium	-27.24	-4.52	-222.61	-78.64	-11.59	1.07	1.25	0.18	<0.02
GM1193	110	Alluv-WCM	-24.60	-4.20	-215.91	-72.21	-10.08	1.07	1.24	0.45	
P7	60.4	WCM	-28.21	-5.00	-310.30	-50.25	-13.04	1.04	1.41	0.70	<0.02
IND3	95.5	WCM	-29.18	-4.28	-215.80	-65.02	-15.87	1.05	1.24	-1.11	
P19	185.9	WCM	-29.65	-5.22	-229.70	-80.11	-9.59	1.08	1.26	-0.15	<0.02
IND1	135	WCM	-31.31	-4.93	-222.10	-70.68	-9.00	1.07	1.25	-0.36	
P4	82.9	WCM	-33.13	-5.27	-209.13	-79.65	-11.28	1.07	1.22	-0.30	
P1	100.9	WCM	-36.13	-5.51	-213.72	-71.84	-6.82	1.07	1.23	0.80	
GM1390	140	WCM	-32.23	-5.32	-211.45	-78.11	-3.48	1.08	1.23	-0.89	<0.02
IND5	114.8	Kumbarilla	-30.11	-4.70	-211.30	-67.80	-10.85	1.06	1.23		
D1	-	CSG	-33.14	-5.16	-209.68	-57.58	10.95	1.07	1.22	-0.62	
D2	-	CSG	-41.29	-6.61	-208.46	-52.59	19.39	1.08	1.21	-1.32	
D3	-	CSG	-39.51	-6.53	-205.37	-50.82	20.43	1.08	1.21		
D4	-	CSG	-40.58	-6.62	-216.00	-50.83	21.32	1.08	1.22	-1.42	
D5	-	CSG	-37.74	-6.01	-211.79	-53.87	18.36	1.08	1.22	-1.21	
D6	-	CSG	-44.07	-7.23	-200.92	-51.61	19.15	1.07	1.20	-1.79	
D7	-	CSG	-42.92	-6.86	-201.99	-50.28	20.21	1.07	1.20	-1.76	
K1	-	CSG	-38.83	-6.13	-197.99	-54.09	13.38	1.07	1.20	-1.03	
K2	-	CSG	-40.81	-6.74	-195.88	-51.12	19.85	1.07	1.19	-2.52	
K3	-	CSG	-36.01	-5.77	-212.30	-55.14	13.82	1.07	1.22	-1.98	
K4	-	CSG	-38.14	-6.21	-206.75	-56.49	9.92	1.07	1.21	-1.24	
K5	-	CSG	-38.08	-6.21	-202.25	-51.54	18.47	1.07	1.21		
K6	-	CSG	-36.50	-5.86	-200.95	-58.31	9.25	1.07	1.21	-0.66	
K7	-	CSG	-39.23	-6.41	-206.43	-53.55	18.99	1.08	1.21	-1.57	
T1	-	CSG	-40.79	-6.57	-200.66	-48.91	22.90	1.08	1.20	-1.17	
T2	-	CSG	-40.90	-6.84	-201.67	-51.85	18.42	1.07	1.20	-0.13	
T3	-	CSG	-40.48	-6.36	-202.05	-49.05	23.03	1.08	1.20	0.36	
T4	-	CSG	-40.69	-6.98	-209.34	-51.99	16.85	1.07	1.21		
T5	-	CSG	-41.40	-6.66	-209.44	-48.86	22.73	1.08	1.21	-0.12	
T6	-	CSG	-41.06	-6.97	-203.79	-50.55	18.18	1.07	1.20		
T7	-	CSG	-40.64	-6.52	-204.92	-50.13	21.62	1.08	1.21	-0.10	

WCM = Walloon Coal Measures. Alluv-WCM = sample at the alluvial basement/alluvial-coal measure interface. CSG = CSG production water sample taken from the relatively deep gas reservoir (200-500 m) in the WCM.

Table S3. Thermodynamic data for all wells with dissolved CH_4 >DL (10 µg/L).

Sample	Depth (m)	Aquifer	[H+]	$[H_2]$	$[HCO_3]$	$[CH_4]$	$[SO_4]$	[HS-]	CO_2 reduction		SO_4 reduction		AOM	
									ΔG^o_T	ΔG	ΔG^o_T	ΔG	ΔG^o_T	ΔG
GM1076	27.7	Alluvium	5.01E-08	1.86E-26	5.94E-03	6.28E-07	3.38E-04	8.71E-07	-229.42	-176.57	-261.88	-94.13	-32.46	-38.44
IND4	35	Alluvium	1.58E-07	1.83E-25	2.84E-03	1.52E-06	2.30E-04	4.99E-07	-229.37	-178.61	-262.05	-102.08	-32.69	-32.39
GM1073	18.3	Alluvium	8.13E-08	4.83E-26	3.67E-03	1.07E-05	7.45E-05	8.32E-07	-229.38	-171.69	-262.00	-93.43	-32.62	-30.42
GM0057	56.7	Alluvium	9.12E-08	6.18E-26	3.99E-03	3.35E-05	1.07E-05	6.61E-07	-229.43	-169.35	-261.86	-90.87	-32.44	-26.57
GM1193	110	Alluv-WCM	6.76E-08	3.37E-26	4.44E-03	1.37E-05	2.13E-04	5.09E-07	-229.40	-170.34	-261.94	-96.21	-32.53	-28.96
P7	60.4	WCM	1.45E-07	1.52E-25	4.86E-03	6.06E-06	1.41E-03	1.12E-06	-229.37	-173.73	-262.03	-104.01	-32.65	-33.76
IND3	95.5	WCM	6.03E-08	2.39E-26	3.28E-03	3.05E-05	6.42E-05	1.84E-05	-229.08	-165.91	-262.97	-77.56	-33.89	-25.18
P19	185.9	WCM	7.76E-09	4.14E-28	5.02E-03	8.75E-05	2.29E-04	2.62E-06	-229.21	-158.21	-262.57	-72.61	-33.36	-34.42
IND1	135	WCM	2.82E-09	5.46E-29	6.91E-03	1.98E-04	4.85E-05	2.84E-06	-229.21	-152.87	-262.56	-60.97	-33.35	-31.56
P4	82.9	WCM	3.47E-08	8.70E-27	3.68E-03	4.31E-04	3.14E-06	2.16E-06	-229.35	-160.24	-262.09	-76.49	-32.74	-28.37
P1	100.9	WCM	1.45E-08	1.54E-27	4.47E-03	7.72E-04	4.65E-06	2.52E-06	-229.40	-156.68	-261.94	-71.61	-32.53	-29.73
GM1390	140	WCM	1.05E-08	8.14E-28	1.45E-02	1.13E-03	4.28E-06	2.46E-06	-229.43	-152.33	-261.86	-69.57	-32.44	-27.56

WCM = Walloon Coal Measures. Alluv-WCM = sample at the alluvial basement/alluvial-coal measure interface. CSG = CSG production water sample taken from the relatively deep gas reservoir (200-500 m) in the WCM.

Table S4. Hydrochemical and ^{3}H for all wells with dissolved CH_4 < DL (10 μg/L).

Sample	Depth	Aquifer	nested site	pH	Temp	DO	TDS	HCO_3	CO_3	SO_4	Cl	Ca	Mg	Na	K	NO_3	Fe total	Mn total	DOC	DOC_{adj}	^{3}H
	m				°C	mg/L	mg/L	meq/L	meq/L	meq/L	meq/L	meq/L	meq/L	meq/L	meq/L	meq/L	mg/L	mg/L	mg/L	mg/L	TU
P3	35.4	Alluvium		7.68	21.1	7.79	866	7.24		0.23	5.92	1.05	1.48	11.04	0.03	3.71E-03	<0.05	<0.001			
GM0119	19.5	Alluvium		7.14	19.93	4.97	656	6.32		0.21	3.35	1.30	1.32	7.61	0.01	1.00E-02	<0.05	0.001			
GM1280	27.8	Alluvium		6.7	21.9	6.79	484	4.58		0.06	2.20	1.25	1.15	4.61	0.01	6.45E-04	<0.05	<0.001			
GM1688	36.75	Alluvium		6.79	23.1	0.67	3760	8.08		2.69	43.38	11.23	7.90	37.74	0.05	1.62E-04	3.31	0.998			
GM0179	18	Alluvium		6.48	18	0.73	17100	6.70		8.21	246.76	50.40	75.11	129.57	0.10	4.84E-04	0.05	0.061	0.90	1.27	<0.02
GM1692	40	Alluvium		6.95	21.2	0.72	3680	6.66		7.85	48.45	14.87	20.07	33.48	0.10	3.23E-04	2.64	0.232	0.10	0.14	<0.02
P6	109	Alluvium		7.37	24.6	3.67	3560	5.96		3.10	38.03	4.09	8.31	39.43	0.15	3.55E-03	0.15	0.013	0.10	0.14	
GM1074	28.9	Alluvium	Dalby	6.64	21.7	2.52	9790	10.60		8.31	147.32	16.17	39.65	95.65	0.13	2.64E-02	<0.05	0.002	0.10	0.14	<0.02
P10	28	Alluvium		6.53	22.5	1.13	3200	6.20		3.44	45.92	8.23	13.57	38.83	0.10	2.32E-02	0.12	0.092	0.50	0.70	
GM1464	79	Alluvium		7.1	19.7	4.23	541	5.82	1.04	0.10	2.17	2.30	2.39	4.09	0.05	2.26E-03	<0.05	0.006	0.20	0.28	<0.02
P18	89	Alluvium		7.47	24	4.93	2760	7.88	1.61	3.73	30.99	4.94	9.46	29.57	0.18	4.03E-03	<0.05	<0.001	0.30	0.41	<0.02
GM1397	24	Alluvium		7.3	24.4	1.84	1260	7.72	1.74	0.21	11.41	2.99	5.27	12.26	0.05	1.53E-02	<0.05	<0.001			
GM1398	24	Alluvium		7.3	23.5	2.39	1630	6.28	1.41	0.23	18.00	3.54	4.52	16.91	0.05	3.64E-02	<0.05	<0.001			
GM1383	31	Alluvium		7.17	21.8	4.20	2110	7.22		0.46	25.55	6.79	12.01	14.70	0.08	1.97E-02	<0.05	<0.001			
GM1173	54.9	Alluvium		7.62	23.1	4.12	717	5.28		0.48	4.79	0.60	1.07	9.04	0.03	5.48E-03	<0.05	<0.001	0.20	0.28	
GM1338	41.2	Alluvium		7.28	21.9	3.81	7860	6.78	0.64	20.58	100.28	7.39	28.05	87.39	0.10	1.18E-02	<0.05	0.041	0.40	0.54	0.22
GM1462	65.5	Alluvium		7.02	22.8	3.50	467	6.04		0.13	1.46	1.90	2.06	3.48	0.05	2.90E-03	0.13	0.003			
P20	76.8	Alluvium	Cecil Plains	7.03	22.6		463	6.04		0.21	0.99	1.95	2.22	3.13	0.05	3.23E-03	<0.05	<0.001	0.30	0.40	<0.02
IND6	52.2	Kumbarilla	Stratheden	7.03	20.3	1.23	5890	2.78		1.48	95.77	26.70	15.71	45.22	0.20	2.10E-03	<0.05	0.030	0.70	0.81	
IND2	84.9	Alluv-WCM	Cecil Plains	7.42	23.7	1.05	890	6.34	1.07	2.83	4.17	1.85	1.73	10.61	0.31	3.23E-04	<0.05	0.464	7.00	9.43	
P2	100.6	WCM		6.71	22.7	1.48	8900	5.36		4.42	131.83	26.50	34.72	74.35	0.23	< 1.62E-04	2.73	0.852	0.30	0.42	<0.02
P5	57.9	WCM		7.7	22.4	2.14	3800	7.18		3.15	40.85	2.25	2.80	50.43	0.15	< 1.62E-04	0.16	0.013	0.50	0.71	
P8	60	WCM		7.19	22.8	5.23	1160	4.84		0.04	11.75	5.94	7.32	6.22	0.03	4.85E-01	<0.05	<0.001	1.20	1.68	
P9	154	WCM		8.28	22.5	2.12	1230	4.48	1.07	1.04	12.23	0.80	0.16	17.52	0.08	9.68E-04	<0.05	0.031			
P12	47	WCM		6.78	23.1	2.08	638	6.32		0.06	2.51	3.29	4.20	3.13	0.13	2.34E-01	<0.05	0.005			0.95
P13	48	WCM		7.05	24.6	10.01	968	7.50		1.19	8.06	4.14	4.94	7.91	0.20	6.29E-03	<0.05	0.008			
P16	89.9	WCM		8.25	23.3	7.87	469	4.18	0.97	0.04	2.31	0.75	0.41	6.00	0.05	1.13E-03	<0.05	0.009			

WCM = Walloon Coal Measures. Alluv-WCM = sample at the alluvial basement/alluvial-coal measure interface. CSG = CSG production water sample taken from the relatively deep gas reservoir (200-500 m) in the WCM. DOC_{adj} calculated as per Peacock et al.[35].

Supporting Information references

1 Whiticar, M. J. Carbon and hydrogen isotope systematics of bacterial formation and oxidation of methane. *Chem. Geol.* **161**, 291-314, doi:http://dx.doi.org/10.1016/S0009-2541(99)00092-3 (1999).

2 Whiticar, M. J., Faber, E. & Schoell, M. Biogenic methane formation in marine and freshwater environments: CO2 reduction vs. acetate fermentation—Isotope evidence. *Geochim. Cosmochim. Acta* **50**, 693-709, doi:http://dx.doi.org/10.1016/0016-7037(86)90346-7 (1986).

3 Clark, I. & Fritz, P. *Environmental Isotopes in Hydrogeology*. (CRC Press LLC, 1997).

4 Paull, C. K. *et al.* in *Proceedings of the Ocean Drilling Program, Scientific Results.* (eds C.K Paull, R Matsumoto, P.J Wallace, & W.P Dillon).

5 Chanton, J. P., Chasar, L. C., Glaser, P. & Siegel, D. in *Stable Isotopes and Biosphere-Atmosphere Interactions, Physiol. Ecol. Ser.* (eds L. B. Flanagan, J. R. Ehleringer, & D. E. Pataki) Ch. 6, 85 – 105 (Elsevier, 2005).

6 Chanton, J. P., Fields, D. & Hines, M. E. Controls on the hydrogen isotopic composition of biogenic methane from high-latitude terrestrial wetlands. *J. Geophys. Res. (G Biogeosci.)* **111**, n/a-n/a, doi:10.1029/2005JG000134 (2006).

7 Botz, R., Pokojski, H.-D., Schmitt, M. & Thomm, M. Carbon isotope fractionation during bacterial methanogenesis by CO2 reduction. *Org. Geochem.* **25**, 255-262, doi:http://dx.doi.org/10.1016/S0146-6380(96)00129-5 (1996).

8 Golding, S. D., Boreham, C. J. & Esterle, J. S. Stable isotope geochemistry of coal bed and shale gas and related production waters: A review. *Int. J. Coal Geol.* **120**, 24-40, doi:http://dx.doi.org/10.1016/j.coal.2013.09.001 (2013).

9 Kinnon, E. C. P., Golding, S. D., Boreham, C. J., Baublys, K. A. & Esterle, J. S. Stable isotope and water quality analysis of coal bed methane production waters and gases from the Bowen Basin, Australia. *Int. J. Coal Geol.* **82**, 219-231, doi:http://dx.doi.org/10.1016/j.coal.2009.10.014 (2010).

10 Flores, R. M., Rice, C. A., Stricker, G. D., Warden, A. & Ellis, M. S. Methanogenic pathways of coal-bed gas in the Powder River Basin, United States: The geologic factor. *Int. J. Coal Geol.* **76**, 52-75, doi:http://dx.doi.org/10.1016/j.coal.2008.02.005 (2008).

11 Rice, C. A., Flores, R. M., Stricker, G. D. & Ellis, M. S. Chemical and stable isotopic evidence for water/rock interaction and biogenic origin of coalbed methane, Fort Union Formation, Powder River Basin, Wyoming and Montana U.S.A. *Int. J. Coal Geol.* **76**, 76-85, doi:10.1016/j.coal.2008.05.002 (2008).

12 McIntosh, J. S., M., Bates, B. in *Technical Workshops for the hydraulic fracturing study: US EPA, Feb 24-25, 2011.* (United States Environmental Protection Agency).

13 Baublys, K. A., Hamilton, S. K., Golding, S. D., Vink, S. & Esterle, J. Microbial controls on the origin and evolution of coal seam gases and production waters of the Walloon Subgroup; Surat Basin, Australia. *Int. J. Coal Geol.* **147–148**, 85-104, doi:http://dx.doi.org/10.1016/j.coal.2015.06.007 (2015).

14 Hamilton, S. K., Golding, S. D., Baublys, K. A. & Esterle, J. S. Stable isotopic and molecular composition of desorbed coal seam gases from the Walloon Subgroup, eastern Surat Basin, Australia. *Int. J. Coal Geol.* **122**, 21-36, doi:http://dx.doi.org/10.1016/j.coal.2013.12.003 (2014).

15 Papendick, S. L. *et al.* Biogenic methane potential for Surat Basin, Queensland coal seams. *Int. J. Coal Geol.* **88**, 123-134, doi:http://dx.doi.org/10.1016/j.coal.2011.09.005 (2011).

16 Draper, J. J. & Boreham, C. J. Geological controls on exploitable coal seam gas distribution in Queensland. *APPEA Journal* **46**, 343–366. (2006).

17 Smith, B. WCR 290. Bungawalbin Creek 1-4 Well Completion Report. PEL13 - Clarence-Moreton Basin (1998).

18 Maher, D. T., Santos, I. R. & Tait, D. R. Mapping Methane and Carbon Dioxide Concentrations and δ13C Values in the Atmosphere of Two Australian Coal Seam Gas Fields. *Water, Air, Soil Pollut.* **225**, 1-9, doi:10.1007/s11270-014-2216-2 (2014).
19 Iverach, C. P. *et al.* Assessing Connectivity Between an Overlying Aquifer and a Coal Seam Gas Resource Using Methane Isotopes, Dissolved Organic Carbon and Tritium. *Sci Rep.* **5**, 15996, doi:10.1038/srep15996 http://www.nature.com/articles/srep15996#supplementary-information (2015).
20 QLD-DNRM. (ed Queensland Department of Natural Resources and Mines) 35 (Queensland Department of Natural Resources and Mines Brisbane, Queensland, 2012).
21 Atkins, M. L., Santos, I. R. & Maher, D. T. Groundwater methane in a potential coal seam gas extraction region. *Journal of Hydrology: Regional Studies* **4, Part B**, 452-471, doi:http://dx.doi.org/10.1016/j.ejrh.2015.06.022 (2015).
22 Warner, N. R. *et al.* Geochemical and isotopic variations in shallow groundwater in areas of the Fayetteville Shale development, north-central Arkansas. *Appl. Geochem.* **35**, 207-220, doi:10.1016/j.apgeochem.2013.04.013 (2013).
23 Hansen, L. K., Jakobsen, R. & Postma, D. Methanogenesis in a shallow sandy aquifer, Rømø, Denmark. *Geochim. Cosmochim. Acta* **65**, 2925-2935, doi:http://dx.doi.org/10.1016/S0016-7037(01)00653-6 (2001).
24 Aravena, R. & Wassenaar, L. I. Dissolved organic carbon and methane in a regional confined aquifer, southern Ontario, Canada: Carbon isotope evidence for associated subsurface sources. *Appl. Geochem.* **8**, 483-493, doi:http://dx.doi.org/10.1016/0883-2927(93)90077-T (1993).
25 Quay, P. D., King, S. L., Lansdown, J. M. & Wilbur, D. O. Isotopic composition of methane released from wetlands: Implications for the increase in atmospheric methane. *Global Biogeochem. Cycles* **2**, 385-397, doi:10.1029/GB002i004p00385 (1988).
26 Moura, J. M. S. *et al.* Spatial and seasonal variations in the stable carbon isotopic composition of methane in stream sediments of eastern Amazonia. *Tellus B* **60**, 21-31, doi:10.1111/j.1600-0889.2007.00322.x (2008).
27 Liptay, K., Chanton, J., Czepiel, P. & Mosher, B. Use of stable isotopes to determine methane oxidation in landfill cover soils. *Journal of Geophysical Research: Atmospheres* **103**, 8243-8250, doi:10.1029/97JD02630 (1998).
28 Chanton, J. P., Rutkowski, C. M. & Mosher, B. Quantifying Methane Oxidation from Landfills Using Stable Isotope Analysis of Downwind Plumes. *Environ. Sci. Technol.* **33**, 3755-3760, doi:10.1021/es9904033 (1999).
29 Bergamaschi, P. *et al.* Stable isotopic signatures (δ13C, δD) of methane from European landfill sites. *Journal of Geophysical Research: Atmospheres* **103**, 8251-8265, doi:10.1029/98JD00105 (1998).
30 Riveros-Iregui, D. A. & King, J. Y. Isotopic evidence of methane oxidation across the surface water-ground water interface. *Wetlands* **28**, 928-937, doi:10.1672/07-191.1 (2008).
31 Tsunogai, U., Yoshida, N. & Gamo, T. Carbon isotopic evidence of methane oxidation through sulfate reduction in sediment beneath cold seep vents on the seafloor at Nankai Trough. *Mar. Geol.* **187**, 145-160, doi:http://dx.doi.org/10.1016/S0025-3227(02)00263-3 (2002).
32 Heilweil, V. M. *et al.* Stream Measurements Locate Thermogenic Methane Fluxes in Groundwater Discharge in an Area of Shale-Gas Development. *Environ. Sci. Technol.* **49**, 4057-4065, doi:10.1021/es503882b (2015).
33 Prinzhofer, A. & Pernaton, É. Isotopically light methane in natural gas: bacterial imprint or diffusive fractionation? *Chem. Geol.* **142**, 193-200, doi:http://dx.doi.org/10.1016/S0009-2541(97)00082-X (1997).
34 Kilgour, B., compiler. National Geoscience Datasets. Canberra: Geoscience Australia. Record 34877, http://www.agso.gov.au/general/technotes/20011023_32.jsp (2001).

35 Peacock, M., Freeman, C., Gauci, V., Lebron, I. & Evans, C. D. Investigations of freezing and cold storage for the analysis of peatland dissolved organic carbon (DOC) and absorbance properties. *Environmental Science: Processes & Impacts* **17**, 1290-1301, doi:10.1039/C5EM00126A (2015).
